# The dynamics of introgression and parallel adaptation across North American *Vitis* species

**DOI:** 10.1073/pnas.2607754123

**Published:** 2026-08-05

**Authors:** Tianpeng Wang, Christopher J. Fiscus, Jacob B. Landis, Noé Cochetel, Abraham Morales-Cruz, Dario Cantu, Jonás A. Aguirre-Liguori, Brandon S. Gaut

**Affiliations:** ^a^ Department of Ecology and Evolutionary Biology, University of California, Irvine, CA 92697; ^b^ School of Integrative Plant Science, Section of Plant Biology, Cornell University, Cornell, NY 14853; ^c^ Department of Viticulture and Enology, University of California, Davis, CA 95616; ^d^ U.S. Department of Energy, Joint Genome Institute, Lawrence Berkeley National Laboratory, Berkeley, CA 94720; ^e^ Genome Center, University of California, Davis, CA 95616; ^f^ Departamento de Ecología Tropical, Campus de Ciencias Biológicas y Agropecuarias, Universidad Autónoma de Yucatán, Merida, Mexico 97100

**Keywords:** adaptive radiation, introgression, phylogenomics, hybrid speciation, grapevines

## Abstract

Hybridization between species can contribute to parallel adaptive events. However, the prevalence of introgression is rarely measured across multiple species, meaning that it is rarely integrated with landscape-scale processes or connected to adaptive repeatability. Here we analyzed whole-genome resequencing data from 639 accessions representing 48 *Vitis* species. *Vitis* is notable for the domesticated grapevine (*Vitis vinifera*), multiple economically important North American species, and as an example of a temperate adaptive radiation. We reconstructed individual- and species-level phylogenetic frameworks for the genus and performed population genetic analyses for 19 species. The analyses uncovered widespread evidence of introgression, comprising ~14% of the average *Vitis* genome. Introgression was associated with the geographic distribution between species, and highly admixed individuals were more frequently found near ecological niche margins. Genetic analyses further indicated that previously recognized hybrid taxa, *Vitis x doaniana* and *Vitis x champinii*, likely represent ongoing hybrid swarms rather than distinct lineages. We assessed patterns of adaptive repeatability across eight species with denser population sampling. For six of eight species, most detected selective sweeps overlapped with sweeps in other species; on average, more recently diverged species shared more overlapping sweeps. A coalescence-based composite likelihood approach inferred that parallel sweep events were driven mostly by introgression, although a substantial fraction (~40%) was attributed to selection on ancestral standing variation. By integrating population genomic data across many species, this study highlights the central role of shared genetic variation—and the driving force of hybridization—across an adaptive radiation.

## Significance

Hybridization is a major force shaping genome evolution, but its role in shaping plant diversification and taxonomic relationships across an adaptive radiation remains unclear. Here we examine hybridization, introgression, and adaptation across the genus *Vitis*, which comprises the domesticated grapevine and economically valuable wild species throughout North America. We find that introgression is widespread, structured by geography and commonly creates hybrid swarms that have not stabilized as distinct species. Instead, introgression fuels adaptive repeatability, which is typified by numerous shared selective sweeps among species. Our results show how gene flow among species can promote repeated adaptation during an adaptive radiation.

Hybridization among species can generate a wide range of evolutionary outcomes, including reinforcement of reproductive isolation (1, 2), formation of hybrid swarms (3–5), lineage collapse (6), and, in rare cases, distinct species (7, 8). Hybridization generally reduces genetic divergence between species or populations, but it can also reassort genetic variants into beneficial combinations that foster adaptation to new ecological niches (9). This reassortment contributes to another major phenomenon: adaptive repeatability.

The study of adaptive repeatability focuses on three major questions (10, 11). The first is whether adaptation occurs in the same genes across different species; when it does, it suggests that genetic solutions to adaptive challenges are constrained. Some evidence supports this view. For example, Whiting et al. (12) performed genome-wide genotype–environment associations across 25 plant species representing divergence times up to >300 Mya. They found that a subset of genes and core physiological pathways have been commonly targeted by natural selection throughout plant evolutionary history (12). A second question is the source, or mechanism, of repeatability. Repeatability between two lineages can be attributed to three mechanisms (10, 11): i) de novo adaptive mutations in separate lineages, ii) selection on variation that segregated in the common ancestor of the two lineages or iii) adaptive introgression (11). The relative frequency of these three mechanisms remains an open question but addressing it will inform our understanding of one potential outcome of hybridization, adaptive introgression. The third question is whether repeatability is a function of divergence time (10). Recently diverged lineages are expected to exhibit more repeatability. This relationship holds across diverse evolutionary lineages (10, 13), but it merits further characterization in plants, given inconsistent results across the few systems studied to date (10).

Population genomic data have yielded remarkable insights into the extent of ongoing and ancient hybridization between species (14). Here we use population genomic analyses to study the patterns and effects of introgression, including its impacts on adaptive repeatability, across the genus *Vitis*. The genus contains ~75 species of perennial vines, with three belonging to subgenus *Muscadinia* (2*n* = 40)(15) and the rest to subgenus *Vitis* (2*n* = 38). Species within subgenus *Vitis* are distributed throughout the temperate Northern Hemisphere, with roughly half native to North America, half to eastern Asia, and one (*Vitis vinifera*) to Eurasia (16). The subgenus is also notable because it represents a temperate adaptive radiation from close tropical relatives (17), making it a plant model for understanding morphological diversity (18, 19) and phylogenetic diversification (17, 20–24). The North American species are particularly important, because they are the source of all viticulture rootstocks (derived from *Vitis riparia*, *Vitis rupestris*, *Vitis berlandieri,* and other species) (25) and because phylogenetic treatments of the genus suggest that North America was the center of species diversification (24). There is evidence for reticulate histories among species (26–28), likely reflecting the fact that all subgenus *Vitis* species are interfertile (29). These reticulate relationships include two taxa of recognized hybrid origin, *Vitis x doaniana* and *Vitis x champinii* (26, 30). *V. x doaniana* is thought to be a hybrid between *Vitis mustangensis* and *Vitis acerifolia*, but the origins of *V.* *x* *champinii* are less clear. One parent is hypothesized to be *V. mustangensis* but the other may be either *V. rupestris* or *Vitis monticola* (30).

For this study we have amassed a whole-genome resequencing (WGR) dataset of >600 accessions representing 48 *Vitis* species, with population genomic sampling for 17 North American species including the two putative hybrids, *V. x doaniana* and *V. x champinii*. We use the dataset to address three sets of questions. First, we characterize genome-wide patterns of admixture and introgression on a genus-wide scale. Is introgression common, and if so, do genomic patterns of introgression correlate across landscape and geographic scales? Second, we investigate taxa of putative hybrid origin. In theory, the genomic history and ages of hybrid groups can be inferred from population genomic analyses (31). What do such analyses reveal about the timing and dynamics of hybridization? Finally, we examine adaptive repeatability. Are shared sweeps common, can they be attributed to a specific evolutionary mechanism, and does their incidence correlate with divergence time? By taking a multispecies, population genomic approach, our work illustrates the varied and extensive role of hybridization across an adaptive radiation.

## Results

### Admixture Is Frequent in *Vitis*.

We called variants for 639 whole-genome samples, including 114 newly generated sequences, belonging to 48 *Vitis* species and 10 outgroup species within the family Vitaceae. The dataset contained 501 samples from 25 North American species that were sampled in the wild, planted as cuttings, and maintained as a living collection at the University of California, Davis. It also included previously published samples from Asian *Vitis* taxa (30 species, 53 samples) (Dataset S1) and a subset of available data for domesticated *V. vinifera* ssp. *vinifera* (44 samples) and its wild progenitor (ssp. *sylvestris*: 39 samples). We mapped the entire dataset against the *Vitis arizonica* v.2.0 genome assembly (32), resulting in mean mapped coverage of 14.6x (Dataset S1). After filtering sites for missing data and poor quality genotype calls, the final dataset consisted of 4,435,884 biallelic SNPs with <5% missing calls and a minor allele frequency ≥0.01 across the entire dataset.

**Fig. 1.**
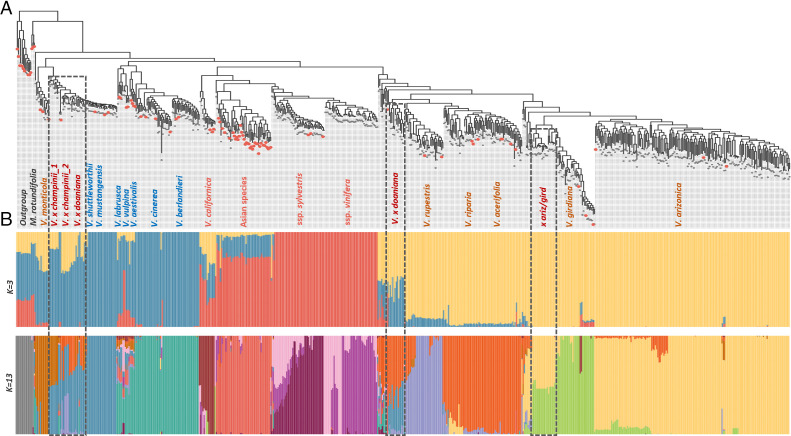
Phylogenomic reconstruction and population structure of 639 accessions. (*A*) The maximum likelihood phylogeny of accessions inferred from genome-wide SNPs. Tips in red indicate the three representative nonhybrid individuals selected per species for species-level phylogenetic inference. Gray rectangles highlight key hybrid lineages (*V. x champinii*, *V. x doaniana,* and a *V. arizonica*/*V. girdiana* complex). (*B*) Model-based ancestry inference across the 639 accessions shown for K = 3 and K = 13. Each vertical bar represents one individual and colors indicate estimated ancestry proportions from the inferred K genetic clusters. Individuals are ordered based on their phylogenetic position. Complete admixture plots for K = 2 to 15 are provided in *SI Appendix*, Fig. S2.

Previous work has shown that the phylogenetics of *Vitis* is complex, likely due to introgression that obscures true species relationships (27). We began our analyses by drafting a phylogenetic tree and examining patterns of ancestry across all accessions. For the latter, we pruned the SNP dataset for linkage disequilibrium (LD) and then inferred ancestry profiles using NGSAdmix (33). An “optimal” number of K = 13 clusters was selected for the 48 species based on log-likelihood and ΔK statistics (Fig. 1 and *SI Appendix*, Figs. S1 and S2). We paired the admixture analysis with the draft phylogenetic tree based on the pruned SNP set (Fig. 1), uncovering widespread evidence for admixture. Some admixture was due to shared ancestry with domesticated *V. vinifera* (34). For example, nearly half of 14 *Vitis californica* samples were admixed with domesticated germplasm, consistent with documented hybridization events between these species (35). Moreover, several *V. arizonica* individuals had ancestry contributions from either *V. rupestris* or *Vitis girdiana,* with some individuals appearing as 50:50 hybrids. Finally, the mixed ancestry of the two putative hybrid taxa (*V. x doaniana* and *V. x champinii*) was evident. *V. x doaniana* comprised two separate clades, reflecting groups with different majority ancestry from the putative parental species (*V. mustangensis* and *V. acerifolia*) (Fig. 1). There were also two distinct sets of *V.* *x* *champinii* samples that differed as to whether *V. rupestris* or *V. monticola* was a primary genetic source.

### Species Phylogeny and Divergence Times.

The study of repeatability requires a phylogenetic framework and divergence time estimates, but extensive admixture among *Vitis* accessions poses challenges for inferring a species phylogeny. To infer such a phylogeny, we first removed samples with substantial admixture (>20%) or that did not cluster as expected with other members of their assigned species (Fig. 1). In total, 140 samples (22% of the dataset) fit one or both of these criteria and were excluded from further phylogenetic and population genomic analyses (*SI Appendix*, Fig. S3 and Dataset S2). Removal of these samples likely makes our population genomic inferences of introgression conservative.

**Fig. 2.**
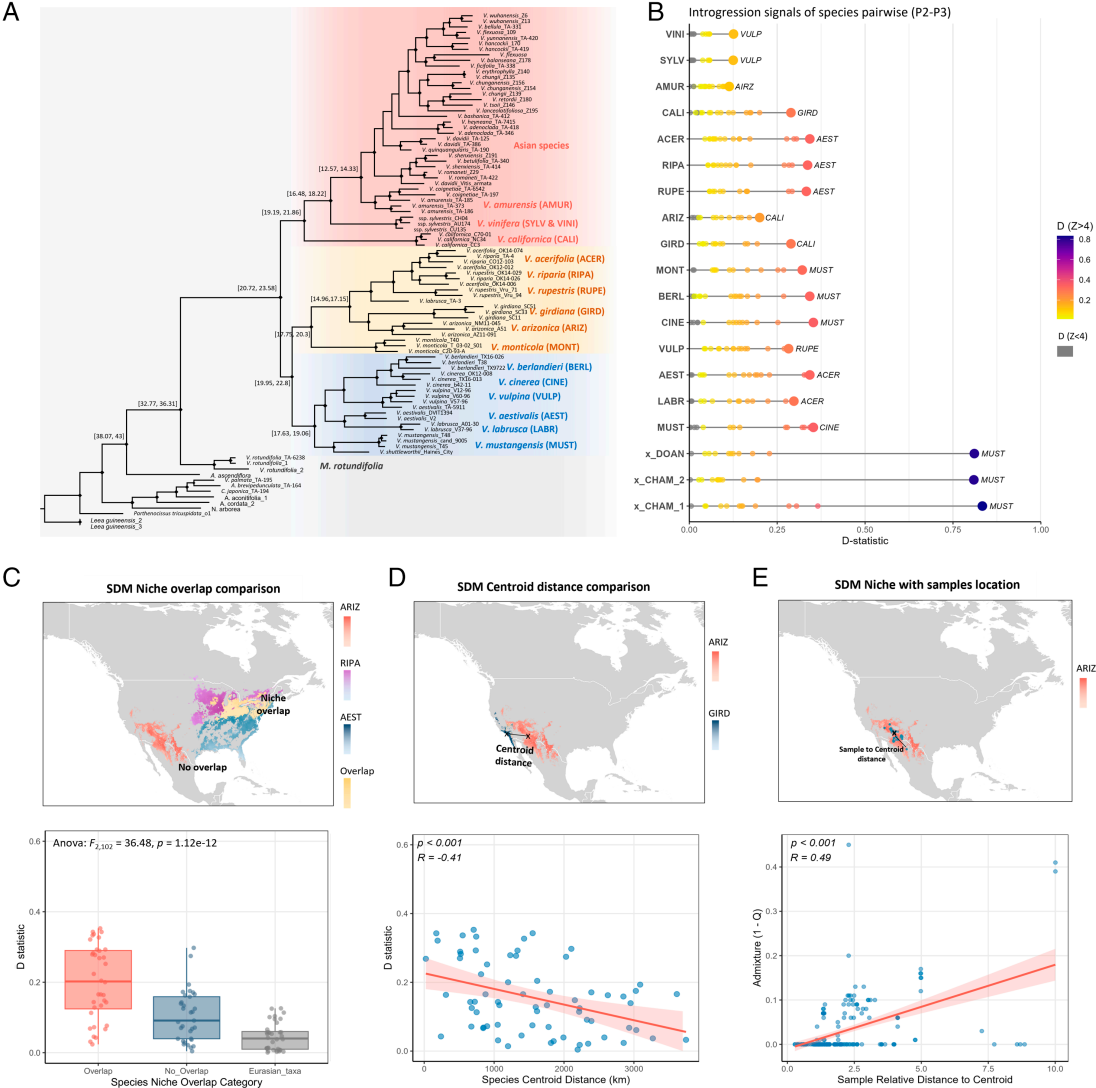
Phylogenetic history, introgression signals, and geographic correlates of gene flow among North American *Vitis*. (*A*) The SVDquartets phylogeny of nonhybrid *Vitis* with branch lengths estimated by maximum likelihood. Node ages were estimated with BEAST2 under a relaxed molecular clock (*SI Appendix*, Fig. S8). Numbers at nodes indicate divergence-time estimates of 95% highest posterior density (HPD) intervals. (*B*) Genome-wide introgression signals from pairwise D-statistics for each P2 species (rows), with points representing various P3 species comparisons. Points are colored by the D-statistics; gray if nonsignificant (Z < 4) and following a gradient for significance *P*-value. The P3 pairing of the maximum D-statistic is labeled for each row. The top three species are Eurasian. (*C*) *Top*: Example species distribution models (SDMs) for *V. arizonica* (ARIZ), *V. riparia* (RIPA), and *V. aestivalis* (AEST) illustrating present-day habitat suitability and niche overlap. *Bottom*: Boxplots summarizing D-statistics for all species pairs (excluding hybrid taxa *V. x doaniana*, *V. x champinii*) that are grouped by SDM niche-overlap category: overlapping North American species pair, nonoverlapping North American species pair, and other Eurasian *Vitis* species. (*D*) *Top*: Example SDM niche centroid-distance comparison for ARIZ and *V. girdiana* (GIRD). *Bottom*: A scatter plot summarizing the relationship between pairwise species D-statistics and centroid distance across all tested species pairs. (*E*) *Top*: Example map shows SDM-predicted niche for ARIZ. *Bottom*: The scatter plot summarizes the relationship, across all individuals and species, between an individual’s admixture level and its location relative to the normalized distance to its SDM centroid. Individual admixture was quantified as 1 − Q, where Q is the individual’s maximum ancestry proportion from population structure at K = 13. Full SDM outputs for all tested North American *Vitis* species are provided in *SI Appendix*, Fig. S12.

We then randomly selected three samples per species where available, producing a dataset of 92 samples across 48 species (Fig. 1 and Dataset S3). With these data, we first constructed phylogenetic trees with RAxML (36) and SVDquartets (37) (Fig. 2*A* and *SI Appendix*, Fig. S5), using LD-pruned SNP genotypes. These two approaches differ in their underlying assumptions about how sequence data relate to a species tree. RAxML infers the maximum-likelihood tree from a concatenated alignment, assuming all sites share a single common history. By contrast, SVDquartets infers the species tree under the multispecies coalescent and is statistically consistent in the presence of incomplete lineage sorting (ILS). Although site concordance factors revealed low (i.e., <40%) site-level support for many branches (*SI Appendix*, Fig. S4), the two trees were highly concordant (Generalized Robinson–Foulds matrix distance = 0.19), discounting the idea that ILS drove the inferred species’ topology. The two trees did differ, however, as to whether the monophyletic Asian clade was nested within the North American clade (*SI Appendix*, Fig. S5). We also assessed the potential effects of reference bias by constructing a third tree, a reference-free tree based on K-mers. The K-mer tree had several rearrangements compared to the previous two (R-F distance = 0.24 from both the RAxML and SVDquartets trees; *SI Appendix*, Fig. S6) but recovered the major clades from the SNP-based trees (*Discussion*).

Taken together, the three phylogenies had at least three consistent and notable features (Fig. 2*A*). First, they supported three major *Vitis* clades corresponding broadly to geographic distributions and ancestry inferred at K = 3 (Figs. 1*B* and 2*A*): the Asian clade, a North American “Western/Central” clade, and a North American “Eastern” clade. The North American “Western/Central” clade contained *V. arizonica* and *V. girdiana,* which are endemic to the Southwest United States (US); *V. rupestris*, which is found primarily in the Ozarks but also in Oklahoma and Texas; and *V. riparia*, which is broadly distributed across the Central and Eastern US (30). The Eastern clade included *V. vulpina*, *V. labrusca*, and *V. aestivalis,* which are found across the Eastern United States; *V. berlandieri* from Texas, New Mexico, and Arkansas; and *V. cinerea* from Florida and Texas (30). Second, the consistency of relationships within clades allowed for divergence time (*t*) estimation (Fig. 2*A* and *SI Appendix*, Fig. S7). We inferred that the *Vitis and Muscadinia* subgenera split ~34 Mya (95% HPD: 32.77 to 36.31 Mya), similar to the age estimated from a smaller sample of complete genomes (38). The subgenus *Vitis* radiation dated to 22.3 Mya (95% HPD: 20.72 to 23.58 Mya), an intermediate value between previous estimates of ~35 Mya (27) and ~18 Mya (38). Third, in all analyses, *V. californica* was sister to the Asian clade, confirming it is the closest North American relative to Asian species (24). We estimated the age of the Asian clade to be 17.7 Mya (95% HPD: 16.48 to 18.22 Mya, Fig. 2*A*), but again our results were inconsistent as to whether the Asian species formed a subclade within North American *Vitis*. As expected, domesticated grapevine and/or its wild relative (ssp. *sylvestris*) defined a separate clade within the Asian group (Figs. 1 and 2*A*). The domesticate was monophyletic, however (Fig. 1), providing no clear evidence for two distinct domestication events (39) with these data despite the inclusion of *sylvestris* samples from throughout its geographic range.

Finally, in addition to nuclear-based trees we also constructed a tree using a common set of 101 plastid genes. As observed previously (24), the plastid-based tree was highly discordant to the three other trees (R-F distance from 0.44 to 0.48; *SI Appendix*, Fig. S8), likely reflecting the inferential limits of a single, uniparentally inherited locus. The tree nonetheless provides some insights into the origin of hybrid groups (see below).

### Population Genomic Signals of Introgression.

To further characterize complex admixture patterns (Fig. 1), we applied population genetic tests for introgression. We limited these analyses to the 19 species for which we had *n* > 3 individuals (Dataset S4), including three species of Asian origin. For each possible pair of species, we computed Patterson’s D-statistic and the related f4-ratio for all possible phylogenetically appropriate species trios (Fig. 2*A*) using *Muscadinia rotundifolia* as the outgroup (Fig. 2*B*). Overall, we tested 144 species pairs for asymmetric allele sharing based on 970 tests (Dataset S5). Of these, 122 of 144 (84.7%) species pairs yielded a maximum D value with a Z-score > 4 (FDR < 0.0001), indicating rampant historical introgression (Fig. 2*B*). Unsurprisingly, the three Eurasian *Vitis* lineages (*V. vinifera*, *V. sylvestris*, and *V. amurensis*) had weak introgression signals with the North American taxa. However, introgression was pervasive among North American species, many of which had evidence for introgression with multiple species. For example, *V. aestivalis* had asymmetric allele sharing with three species in a separate clade (*V. riparia*, *V. rupestris,* and *V. acerifolia*), perhaps reflecting gene flow prior to the divergence of the three species. The f4-ratio results were similar but provided an estimate of the admixture proportion for each species (*SI Appendix*, Fig. S9). Across 19 species, the average f4-ratio was 0.14 (range: 0.01 to 0.78), meaning that on average roughly 14% of the genomes across our sample could be attributed to introgression. Consistent with their putative hybrid origins, *V. x doaniana* and *V. x champinii* had the highest D-statistics (D > 0.75; Fig. 2*B*) and highly elevated f4-ratios (*SI Appendix*, Fig. S9); when they were removed from the dataset, the average f4-ratio was 0.12.

D-statistics are correlated across species pairs due to shared phylogenetic histories, so we explored alternative approaches to explore the history of introgression. One was TreeMix, which also revealed pervasive introgression, including historical introgression events between *V. aestivalis* and the *V. acerifolia/riparia/rupestris* clade, as well as between the *V. arizonica/girdiana* clade and the *V. acerifolia/riparia* clade (*SI Appendix*, Fig. S10). We also applied the f-branch (fb) statistic, which assigns excess allele sharing to specific branches on a species phylogeny. This analysis identified 42 branches with significantly elevated fb signals (fb > 0.1; FDR < 0.0001), including both external and internal branches. Inferred introgression events included *V. arizonica* with *V. monticola*, *V. labrusca* with *V. vulpina*, and *V. californica* with both *V. arizonica* and *V. girdiana*. The strongest fb signals again confirmed the hybrid status of *V. x doaniana* and *V. x champinii* (*SI Appendix*, Fig. S11).

### Genomic Introgression Signals Correlate with Geography.

Our study integrates across many species, making it possible to assess patterns that would be more challenging with just a few species. For example, it seems reasonable to hypothesize that geographically closely related species introgress more often. To test this idea, we used species distribution models (SDMs) to estimate the geographic range of 14 North American species, excluding the putative hybrid taxa (*V. x doaniana* and *V. x champinii*) to avoid confounding species-level patterns (*SI Appendix*, Fig. S12). We first identified which species pairs have overlapping geographic distributions and plotted the distribution of D statistics for overlapping species compared to nonoverlapping species. Overlapping species pairs had higher average D statistics (ANOVA: *F*_2,102_ = 36.48, *P* = 1.12e-12; Fig. 2*C*). Second, we calculated the geographic centroid of each SDM and then, for each species pair, calculated the geographic distance (*d*_centroid_) between centroids. The pairwise D and *d*_centroid_ matrices were correlated, too, based on both multiple regression matrix analysis (*P* < 0.001) and linear regression analysis (Pearson’s *r* = −0.41; *P* =2.35e-5; Fig. 2*D*).

We also explored the hypothesis that introgression facilitates adaptation to new ecological niches (9). We predicted that introgression should be more evident at geographic edges, where habitat tends to be more marginal and where the opportunity for hybridization may be more frequent. To test this idea, we used the inferred admixture proportions of each individual (Fig. 1), the sampling location of each individual when available (Dataset S4), and the location of each individual relative to the geographic centroid of its assigned species. After combining data across 233 individuals with location information that represent eight species, we found a significant positive correlation (Pearson’s *r* = 0.49; *P* = 2.12e-15) between the admixture proportion of individuals (as measured by 1 − *Q*, where *Q* is an individual’s maximum ancestry proportion based on the admixture analyses; Fig. 1) and their normalized distances from geographic centroids. The strength of the relationship was diluted by including species with little evidence for introgression; analyses on a per species basis yielded higher correlations for several species (e.g., *V. arizonica and V. rupestris*), with one as high as *R* = 0.70 (*SI Appendix*, Fig. S13). Altogether, these results show that *Vitis* has been profoundly shaped by widespread introgression, that introgression between species scales with geographic distance, and that these patterns are consistent with more admixture at geographic edges.

### The Genomic History of Hybrid Taxa.

Our analyses consistently uncovered strong evidence of admixture within *V. x doaniana* and *V. x champinii*, reflecting their hybrid origins (26, 30) (Figs. 1 and 2*B*). However, population genetic data can provide further insights, such as whether they appear to be recent hybrid swarms or perhaps historically stable lineages. To investigate the history of hybrid groups, we performed a series of population genomic analyses on hybrids and their parental donors. For simplicity, we present results based on *V. x doaniana* here, with parallel analyses reported in the supplement for the two distinct *V. x champinii* groups and another hybrid group of *V. arizonica*/*V. girdiana* individuals.

**Fig. 3.**
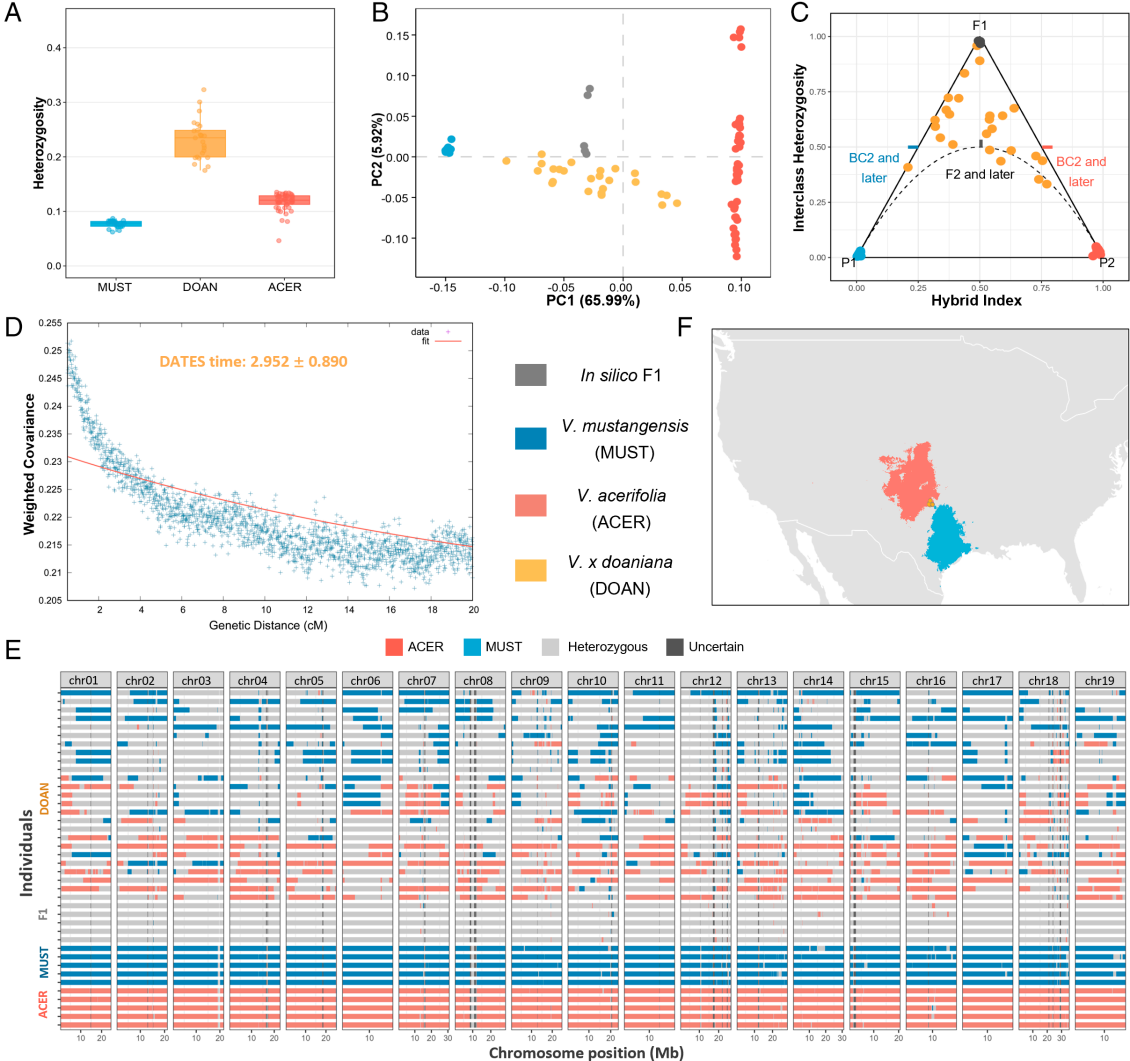
Genomic evidence for the recent hybrid origin of *Vitis x doaniana*. (*A*) Comparison of genome-wide heterozygosity for *V. x doaniana* and the hypothesized parents (*V. mustangensis* and *V. acerifolia*). (*B*) PCA of whole-genome SNPs of the three parental and hybrid groups along with in silico F1 individuals. (*C*) Triangular plot of hybrid index (*S*) vs. interclass heterozygosity (*H*). The plot compares natural *V. x doaniana* samples against diagnostic markers from parental species and in silico F1 controls, classifying individuals as early-generation hybrids (F1/F2) or backcrosses. The points above the dotted curve represent individuals that are estimated to be backcross 2 (BC2) individuals or more recent. (*D*) Inference of admixture generation timing using DATES. The curve displays the decay of weighted ancestry covariance against genetic distance (cM) for *V. x doaniana* populations. The average inferred age is 2.9 generations. (*E*) Chromosome painting visualization using WinPCA results of ancestry assignment. Each row represents the 19 chromosomes of a single individual. Genomic windows are colored by ancestry state: homozygous for *V. mustangensis* (blue), homozygous for *V. acerifolia* (red), or heterozygous (gray). (*F*) Geographic distribution showing complete overlap between the location of sampled *V. x doaniana* individuals and the species niche of one parent (ACER).

We began by performing ADMIXTURE (K = 2) with *V. x doaniana* and its two parental species (*V. mustangensis* and *V. acerifolia*) (*SI Appendix*, Fig. S14), by measuring genome-wide heterozygosity for the parent–offspring trio (Fig. 3*A*) and by applying principal component analysis (PCA) to the genomic data (Fig. 3*B*). To aid the interpretation of the PCA and additional analyses, we included artificial “controls”—i.e., in silico F1 hybrids produced by combining SNP variants from parental individuals (*Materials and Methods*). The results showed that heterozygosity of *V. x doaniana* was higher than either parent, suggesting the possibility of recent hybridization; further, both PCA and admixture had *V. x doaniana* individuals as intermediate between donor species. We used triangular plots—which visualize ancestry proportions among three taxa (40)—to evaluate these signals more precisely. As expected, the in silico F1 hybrids were placed at the apex of the plot, while the *V. x doaniana* individuals were largely aligned with the in silico hybrids or with regions consistent with early-generation backcrosses (Fig. 3*C*). The shallow age of these hybrids was further supported by DATES analyses, which estimates the timing of hybridization events for a single individual based on the size of recombination blocks (Fig. 3*D*). DATES estimated that the hybridization age of the 20 *V. x doaniana* individuals ranged from 1.58 ± 0.73 to 16.56 ± 4.83 generations, with an average of 2.95 ± 0.89 generations (*SI Appendix*, Fig. S15). Chromosome painting for each individual based on winPCA (41) revealed large, unbroken chromosomal segments from each parental species (Fig. 3*E*) as did topology weighting analyses (*SI Appendix*, Fig. S16), a pattern consistent with recent hybridization and limited recombination. We also investigated chloroplast haplotypes of hybrid individuals. *V. x doaniana* individuals were not monophyletic, and individuals clustered phylogenetically with both parents, suggesting bidirectional hybridization (*SI Appendix*, Fig. S17). Finally, we considered whether geography and ecology make *V. x doaniana* distinct from its parents, but all sampled *V. x doaniana* individuals were localized within the *V. acerifolia* SDM (Fig. 3*F*). Altogether, these observations suggest that *V. x doaniana* is a recent bidirectional hybrid without a distinct geographic distribution, at least at the resolution of our analyses. We uncovered qualitatively identical results for the recent origin of the three other hybrid groups (*SI Appendix*, Fig. S18).

### Extensive Repeated Adaptation between *Vitis* Species.

**Fig. 4.**
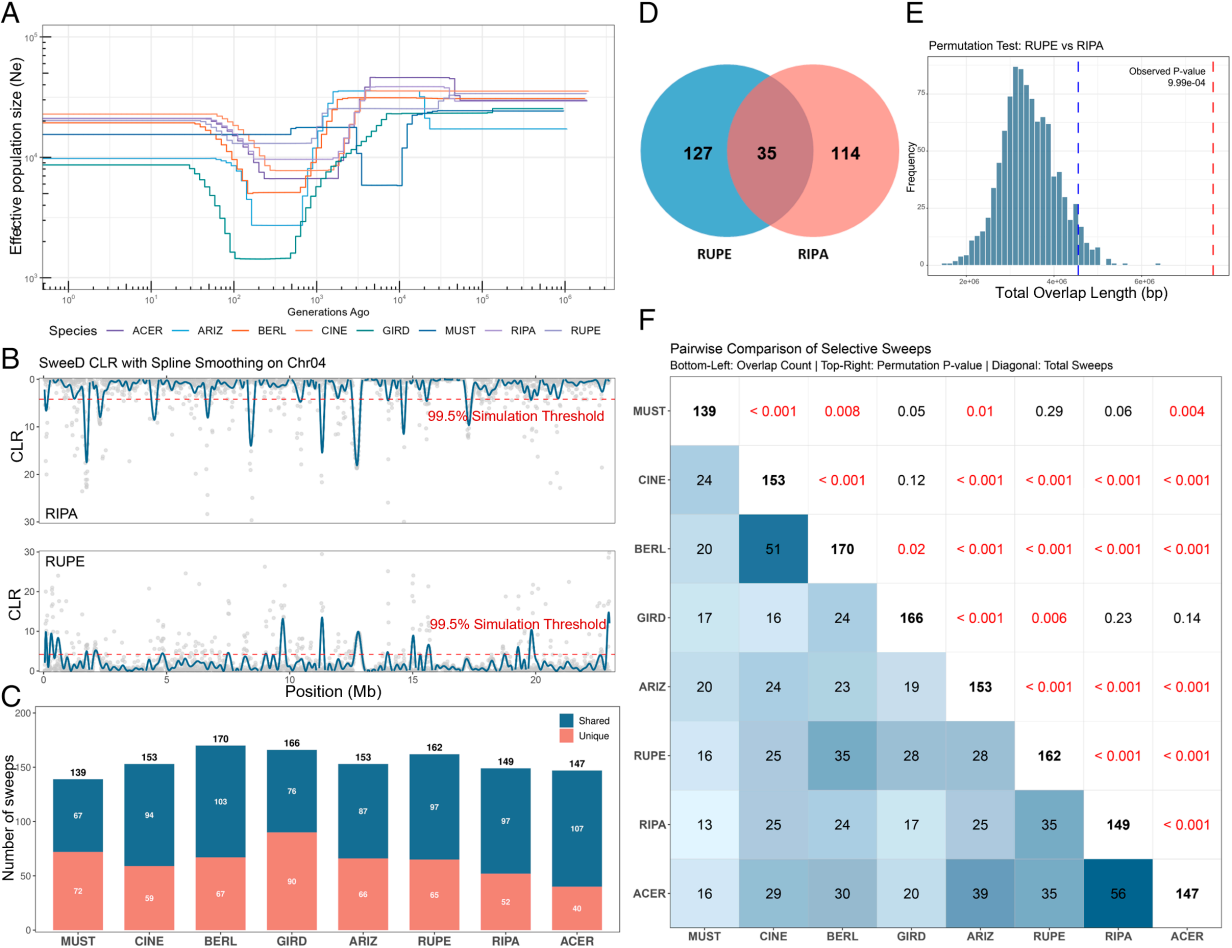
Identification and pairwise comparison of convergent selective sweeps among eight North American *Vitis* species. (*A*) Demographic history trajectories inferred for selected *Vitis* species, using mushi. The plot displays changes in effective population size over time (generations ago). (*B*) Examples of genome-wide scan for selective sweeps in species pair *V. riparia* (RIPA) and *V. rupestris* (RUPE) on chromosome 4. The solid blue line represents the spline-smoothed CLR profile used to identify peaks. The horizontal red dashed line marks the significance threshold, defined as the 99.5th percentile of neutral coalescent simulations. (*C*) Counts of unique and shared selective sweeps identified for each species. (*D*) Venn diagram showing the number of unique and shared selective sweeps between example species pair *V. rupestris* (RUPE) and *V. riparia* (RIPA). (*E*) Permutation test assessing the significance of putative sweep overlaps between RUPE and RIPA. The histogram shows the null distribution of overlap lengths expected by chance. The vertical red line indicates the observed overlap (*P* < 0.001); the vertical blue line represents *P* < 0.05. (*F*) Pairwise summary matrix of selective sweeps across eight *Vitis* species. Diagonal (bold): Total number of significant selective sweeps identified for each species. Lower Triangle (blue heatmap): The count of shared sweep regions between each species pair. Upper Triangle: *P*-values from permutation tests assessing whether the observed sharing is significantly greater than random expectation. Significant FDR-adjusted *P*-values (<0.05) are highlighted in red.

Our multispecies sampling provides a unique opportunity to assess the repeatability of adaptive evolution, specifically whether adaptive evolution has targeted the same genomic regions across species. To explore these ideas, we focused on eight North American species with *n* ≥ 15 samples *(V. mustangensis, V. cinerea, V. berlandieri, V. girdiana, V. arizonica, V. rupestris, V. riparia, and V. acerifolia)*, reflecting the fact that higher sample sizes provide more accurate inferences of selective sweep positions (42). We scanned each species for signatures of recent positive selection using the composite likelihood ratio (CLR) statistic. To differentiate adaptive signals from demographic noise, we established significance thresholds for each species using neutral coalescent simulations that incorporated species-specific demographic histories (Fig. 4*A* and *SI Appendix*, Figs. S19 and S20). After we identified putative selective sweeps in each species, we defined a selective sweep as “shared” between species if the genomic intervals overlapped by > 60% of the sweep length.

We identified numerous regions consistent with positive selection (Fig. 4*B*). The total number of significant selective sweeps varied from 139 in *V. mustangensis* to 170 in *V. berlandieri*, representing between 5.06 and 7.13% of the total genome. The average sweep length was 174.5 kb, with 95% of sweeps between 23.5 kb and 505.7 kb (Fig. 4*F* and *SI Appendix*, Fig. S21 and Dataset S6). Each species contained unique (i.e., nonshared) sweeps, ranging from 40 in *V. acerifolia* to 90 in *V. girdiana*, but for six of eight species the majority of sweeps overlapped with those from other species (Fig. 4*C*). Examining the total of 734 shared sweeps, most overlapped between two (340) or three (213) taxa, and only one was shared among seven or more species (*SI Appendix*, Fig. S22).

We performed permutation tests to test whether sweeps were shared more often than expected by chance between species. The tests shuffled the locations of sweeps randomly across genomes while preserving their size. We illustrate the approach with *V. rupestris and V. riparia*, which shared 35 sweeps (Fig. 4*D*). After generating null distributions from shuffled sweeps, the observed length of overlapping sweeps was far greater than expected by chance (*P* < 0.001; Fig. 4*E* and *SI Appendix*, Fig. S23). We repeated this analysis across all species pairs, revealing a pattern of repeated adaptation, with 22 of 28 pairwise comparisons significant (FDR-adjusted *P* < 0.05) (Fig. 4*F*). Not surprisingly, the strongest signals of repeatability were observed between phylogenetically close or geographically overlapping lineages—e.g., the *V. cinerea–V. berlandieri* pair and the *V. riparia–V. acerifolia* pair. Many of the nonsignificant comparisons included more distantly related or largely allopatric species, such as *V. girdiana*–*V. riparia*. Finally, we examined gene content in sweeps, finding that a small proportion (2.66% of 734) contained no genes. Combining genes from putative sweeps across all eight species, GO analyses uncovered 34 significantly (*P* < 0.05) enriched categories (*SI Appendix*, Fig. S24). As is often the case with GO enrichment analyses, there was no compelling signal, but enrichment categories included terms related to defense responses and to responses to ion concentrations that could be indicative of environmental adaptation.

### Introgression Is the Dominant Mode of Repeated Adaptation.

**Fig. 5.**
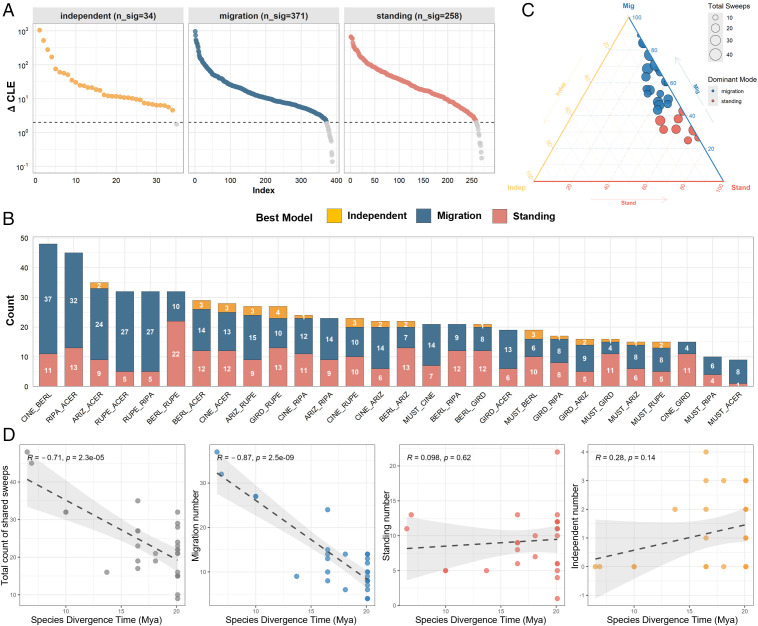
Inference of evolutionary mechanisms driving convergent adaptation in *Vitis*. (*A*) Assessment of model support for three modes of convergence: independent mutation, migration, and standing variation (i.e., ancestral sorting). The plots display the difference in composite log-likelihood between the best-fitting convergent model and the neutral model for each shared sweep region. The dashed horizontal line marks the significance threshold (ΔCLE > 2); only regions exceeding this threshold were considered to have significant support and were retained for downstream analysis. (*B*) Distribution of the best-supported evolutionary models across species pairs. The counts of shared sweeps best explained by migration (blue) and standing variation (red) far exceed those explained by independent de novo mutation (orange). (*C*) Ternary plot summarizing the proportion of shared sweeps assigned to each evolutionary mode for each species pair. Mig = migration model; Indep = de novo mutation model; Stand = standing variation model. (*D*) Correlations between divergence time (x-axis) and the convergence mechanism based on (from left to right) the number of total shared sweeps, migration sweeps, standing-variation sweeps, and independent-mutation sweeps.

The significant excess of shared selective sweeps among *Vitis* species raises at least two questions: does repeatability arise from independent de novo mutations, selection on ancestral standing variation, or adaptive introgression? And how do these mechanisms vary as a function of divergence time? To distinguish among the three mechanisms, we applied a coalescent-based composite likelihood approach that models the probability of shared sweep signatures under the three distinct evolutionary mechanisms (43). For each shared sweep region identified in our *Vitis* species pairwise comparisons, we computed the likelihood of i) neutrality, ii) independent de novo mutation, iii) migration (i.e., adaptive introgression), and *iv*) selection from ancestral, standing variation. We retained only those regions where the best-fitting adaptive model significantly outperformed the neutral model (composite likelihood scores difference, ΔCLE > 2), reducing the total number of shared sweeps to 663 from 734 (Fig. 5*A*). The analysis of these 663 shared sweeps suggested that convergent adaptation is rarely fueled by independent mutation; across all species pairs, only ~5% (34 of 663) of shared sweeps were best explained by the model of independent de novo mutations (Fig. 5*A*). In contrast, 56% and 39% of shared sweeps better fit the adaptive introgression and standing variation models (Fig. 5 *A* and *B*). It is worth emphasizing that these results were not due to the contribution of just a few species pairs, because migration was the numerically dominant model in 18 of 28 pairwise species comparisons (Fig. 5*C*).

We investigated whether repeated evolution scaled with divergence time (*t*) (10) by plotting the number of shared sweeps for each species pair against *t* estimates (Fig. 2*A* and *SI Appendix*, Fig. S8). The resulting plot revealed a strong negative correlation (*R* = −0.71; *P* = 2.3e-5; Fig. 5*D*), and significance was confirmed by phylogenetic generalized linear mixed model (PGLMM) analysis (*P* = 4.4e-4). We also plotted each of the three categories separately. There was no statistically supported relationship between *t* and either the number of de novo sweeps or the number of sweeps based on ancestral variation. In contrast, the number of adaptive introgression events declined over time (linear regression, *R* = −0.87, *P* = 2.5 × 10^−9^; PGLMM, *P* = 4.0e-5), but it did not drop to zero. Interestingly, even species that diverged at the origin of the subgenus ~22 Mya shared overlapping sweeps (Fig. 5*D*).

## Discussion

Population genomic datasets spanning multiple species can simultaneously inform about phylogenetic relationships, divergence times, hybridization, the genomic basis of trait evolution and even landscape-scale evolutionary patterns (44, 45). Datasets of this type are likely to be particularly informative in plants, where hybridization has long been proposed as a driver of adaptive radiation (4) and may affect species’ relationships. Despite its importance, few plant systems currently offer whole-genome resequencing data that include population sampling across multiple species. Our *Vitis* dataset thus represents a rare opportunity to explore the effects of introgression on adaptive repeatability.

### Vitis Species’ Relationships: An Emerging Consensus.

Accurate phylogenetic inference is foundational for evolutionary analyses; in our case, a phylogenetic framework supported the estimation of divergence times, which in turn allowed us to evaluate the pace of adaptive repeatability (see below). *Vitis* has been a frequent target for phylogenetic analysis (17, 20–24), but phylogenetic treatments have generally failed to reach a consensus about *Vitis* species’ relationships (46). One overarching cause for the lack of consensus is likely to be the rampant introgression that we have documented here. Another cause of phylogenetic inconsistencies has been previous reliance on plastid markers, given well-known phylogenetic discordances between plastid and nuclear trees (47) (*SI Appendix*, Fig. S8). Additional reasons include inconsistent species sampling across datasets, which hamper comparisons, and limited sampling (i.e., often one sample) from each species. The latter is an issue because a single sampled individual may inadvertently reflect an admixture event more than species’ history.

There is no simple solution to assess species relationships in the presence of reticulate evolution. Here we chose to generate a total evidence tree based on all available data (Fig. 1) and constructed species-level trees after filtering notably admixed individuals (Fig. 2*A* and *SI Appendix*, Figs. S5–S7). The primary intent of our filtering step was to remove individuals of ambiguous genetic provenance while retaining some within-species sampling. This filtering step may, however, make species’ relationships appear more distinct than they should be, given introgression and ILS. To help evaluate the potential effects of ILS, we applied two methods (RAxML, SVDquartets) that differ in their assumptions about ILS. We also built a K-mer tree to avoid reference biases. These three treatments yield largely consistent trees (*SI Appendix*, Figs. S5–S7). The phylogenetic inferences based on our three trees also mirror those from an independent study based on nuclear data from 43 species (24), which identified nine major clades. Although their species sampling was not identical, our trees recapitulated their nine main groups as well as most of the relationships among groups. The most lingering uncertainty pertains to the origin of the Asian clade, which varied among our trees and also among previous phylogenetic studies. Nonetheless, the concordance between our and previous trees (24) is beginning to yield a consensus for *Vitis*; a robust phylogenetic framework is emerging despite extensive historical and recent introgression.

### Ancient and Ongoing Introgression Is Common.

The prevalence of introgression was evident from signals of asymmetric allele sharing across the genus, based on multiple metrics (Fig. 2*B*). Patterson’s D-statistic identified significant asymmetric allele sharing in 84.7% of 144 species pairs, and the f4-ratio metric estimated admixture proportions averaging 0.14 across 19 species samples. TreeMix similarly supported extensive historical gene flow, while f-branch analyses localized excess allele sharing to dozens of external and internal branches on the species phylogeny (Fig. 2*A* and *SI Appendix*, Figs. S9–S11). These results extend previous work in *Vitis* (26–28, 48) and drive home that introgression has not only been common but pervasive.

The pattern of introgression is not random, however, because it is structured by geography and ecology, as measured by bioclimatic variables integrated into SDMs. For example, we found that the magnitude of D statistics between species pairs was elevated for species that overlapped in geographic niche space and correlated negatively with geographic distances (*d*_centroid_) between species (Fig. 2 *C* and *D* and *SI Appendix*, Fig. S12). Surveys of suboscine birds and wild tomatoes have also found D-based introgression signals to be higher for species in close geographic proximity (49, 50). It is difficult to conclude that geography is causal, however, since introgression is likely to be confounded with other factors like genetic distance (51) and phylogenetic relationships. At the individual level, admixed individuals are more likely to be located at species’ margins (Fig. 2*E* and *SI Appendix*, Fig. S13), a result consistent with introgression facilitating persistence in marginal habitats at the edge of species’ distribution (52). Similar phenomena have been documented in *Quercus* (oaks) (53, 54), *Populus* (poplars) (55) and *Carex* (56). It is worth noting, however, that effects at the edge of species’ distributions are likely to be nuanced. For example, a study of *V. arizonica* found that populations at the edge of range expansion have more deleterious alleles and fewer adaptive alleles than geographically central populations, likely due to dispersion and small population sizes (57). Our point is that introgression may aid persistence and adaptation at species’ edges, but it may also be insufficient to erase genome-wide signatures associated with range expansion and marginal habitats.

Our results also illustrate that introgression has been not only historical but recent and ongoing, based in part on our analysis of hybrid groups. For the four hybrid groups we tested, all genomic evidence points to their recent origin. Ancestry-based approaches placed hybrid individuals as similar to in silico F1 controls, triangular plots were consistent with early-generation backcrosses, and estimated ages of individuals were recent (i.e., from 2 to 17 generations, representing roughly <10 to 60 y) (Fig. 3 and *SI Appendix*, Fig. S15). Chromosome painting also revealed large, unbroken ancestry tracts, which is consistent with limited opportunity for recombination since admixture (Fig. 3*E*). These results contrast markedly with genomic patterns from established, distinct hybrid species (58–62). For example, the genomes of *Senecio squalidus*, which is only ~300 y old, display evidence of fragmented ancestry and reduced heterozygosity (63). To become distinct and stable, a hybrid lineage needs to evolve some degree of reproductive isolation from its parents or occupy a distinct ecological niche that promotes persistence (31, 64), but the common use of both taxa in grapevine genetic analyses and breeding (e.g., ref. 65) suggests there is little, if any, reproductive isolation. Further, our sampled *V. x doaniana* individuals fell within the SDM of *V. acerifolia* (Fig. 3*F*), so it is not ecologically distinct at this scale. Our results do, however, shed light on the disagreement over the parentage of *V. x champinii* (30), because there are two groups: one has *V. rupestris* parentage while the second has *V. monticola* (Fig. 1).

### Introgression Fuels Repeated Adaptation.

Rampant hybridization can lead to adaptive introgression that manifests as repeatability. Recent studies have measured adaptive repeatability in plant systems using one of two approaches. The first is to test for genotype–environmental associations and assess whether homologous/orthologous genes exhibit associations across different lineages (12, 66, 67). The second, which we have used here, is to compare regions of putative selective sweeps among species. The advantages of this approach include an explicit genomic context and the ability to detect phenomena outside coding regions. Relevant examples come from studies in *Zea*, where there is evidence of parallel selective sweeps across subspecies and populations (68, 69). Note, however, that the genus *Zea* radiated only ~150,000 y ago (70, 71), representing at best “a blink of an eye” on an evolutionary timescale.

*Vitis* is a suitable model for studying repeatability because it is speciose, has highly collinear genomes across species (38), and represents an appreciable expanse of evolutionary time—i.e., ~34 My (Fig. 2*A* and *SI Appendix*, Fig. S8). Our analysis of selective sweeps across North American *Vitis* extends previous research (28) by increasing the number of species, improving the specificity of selective sweep mapping by explicitly incorporating demographic inference, and focusing explicitly on potential mechanisms of repeatability. Most species pairs (22 of 28 = 78%) share significantly more selective sweeps than expected by chance (Fig. 4), suggesting extensive parallel evolution. The interpretation of 78% must be tempered by understanding that the statistical power to detect sweeps varies among species, that there is statistical nonindependence across species pairs, and that our genome-wide permutations could be anticonservative if sweeps are limited to just a few gene-rich or recombination-poor genomic regions. We believe it unlikely, however, that genome architecture alone accounts for the striking degree of parallelism. One reason is that ~35% of the reference grapevine genome is gene rich (72), meaning the proportion of genomes encompassed in sweeps is still small compared to the gene rich region. Furthermore, if shared sweeps are caused by genomic architecture, one expects sweeps to be shared across most or all taxa, reflecting the highly conserved architecture across *Vitis* genomes (38). However, only one of 734 putative sweeps overlapped across > 6 species, while 75% overlapped between only two or three species (*SI Appendix*, Fig. S22).

The high degree of shared sweeps raises the question of mechanism: does repeatability arise from independent de novo mutations or from shared pools of genetic variation? A coalescent-based modeling framework (DMC) (43, 73) documented overwhelming support for models of shared ancestry (Fig. 5). Specifically, we identified 629 shared adaptive regions that were better explained by the two shared ancestry models—adaptive migration or ancestral sorting—compared to only 34 attributable to independent mutations (Fig. 5*A*). Among shared ancestry models, our analyses estimate that repeatability occurs ~1.45-fold more often via adaptive introgression (i.e., migration) than through ancestral sorting. This general pattern aligns with *Zea*, where selective sweeps were shared among populations most frequently via migration (69). It must be remembered, however, that our sweeps were categorized by the model with the most support, as opposed to statistically significant differences in model likelihoods. For example, among the 663 sweeps that favored the nonneutral models, 140 did not show statistically significant support (i.e., ΔCLE < 2) for the best-fitting model over the second-best alternative. There is thus some uncertainty in model categorization. Nonetheless, the inescapable conclusion is that shared ancestry is the principal driver of adaptive repeatability in *Vitis*, and the evidence points to adaptive introgression as the most common source for parallel events.

The drivers of shared ancestry are expected to dissipate over time (10), because more distant lineages should share fewer segregating ancestral variants and/or have higher impediments to gene flow due to either geographic or reproductive isolation (10, 13). We tested this idea based on *Vitis* species that ranged in divergence time from 6.5 to 22 Mya (*SI Appendix*, Fig. S8). There was a strong negative relationship between *t* and the number of shared sweeps; the relationship was particularly pronounced for sweeps attributed to migration (Fig. 5*D*). These observations expand our understanding of the pace and distribution of repeatable adaptive events and suggest that *Vitis* adaptation follows a “repeated sorting” framework, where traits evolve in related lineages via the selection of identical-by-descent alleles (11, 74). For North American *Vitis*, it is clear that “reinventing the wheel” from de novo mutation is evolutionarily inefficient compared to borrowing (via introgression) or inheriting (from standing variation).

### Synthesizing the Effects of Introgression: The Syngameon Hypothesis.

Taken together, our results show that introgression in *Vitis* is widespread on geographic and temporal scales. Geographically, introgression is more common among overlapping or nearby species and is elevated near niche margins (Fig. 2 *C*–*E*). Temporally, hybrid swarms suggest recent hybridization, as does admixture between *V. californica* and cultivated grapevine (Fig. 1), given the recent arrival of *V. vinifera* in the western United States. Yet introgression in *Vitis* is not solely a contemporary phenomenon, because TreeMix and *f-*branch statistics infer migration edges on internal branches deep in the species tree (*SI Appendix*, Figs. S10 and S11) and DMC analyses detect adaptive introgression between species that diverged >20 Mya (Fig. 5*D*). These results portray a continuum of introgression that has operated throughout the *Vitis* radiation and continues to shape contemporary populations.

These observations raise a question: given genetic exchange across time and space, how does *Vitis* maintain distinct species? Despite pervasive admixture, the species retain diagnostic leaf morphologies (75), growth habits, and ecological preferences (19, 30). Moreover, our phylogenomic analyses recover the same major clades identified by an independent study (24) even though, on average, ~14% of each genome is introgressed. This pattern—i.e., extensive gene flow without lineage collapse—is a potential signature of porous species boundaries, where selection maintains differentiation at loci underlying ecological or reproductive divergence while the genomic background exchanges more freely (76–78). This pattern is also a defining feature of syngameons, which are networks of species connected by gene flow that retain distinct phenotypic and ecological identities (78–80).

The syngameon concept is perhaps best illustrated by *Quercus*, where genus-wide introgression coexists with stable species across heterogeneous landscapes (53, 80). Similar patterns have been described in pines (78), *Populus* (55), *Carex* (56), and other species. *Vitis* shares several life-history features with these syngameon groups, including long generation times, broad sympatry, generalist insect or wind pollination, and weak reproductive boundaries, all of which lower barriers to interspecific gene flow (81). Hybridization in *Vitis* is bolstered by the presence of extensive contact zones; even species from distinct phylogenetic clades—such as the “Central/Western” clade and the “Eastern” clade—have spatial overlap (*SI Appendix*, Fig. S12). Moreover, studies of complete genomes throughout the subgenus have discovered few of the genomic features, like prominent inversions or extensive variability in genome size, that might impede introgression on a genomic level (38). The syngameon framework also aligns with our observation that hybrid swarms are common, because hybrid swarms define active nodes in a syngameon network (78). Hybrid swarms can reinforce species boundaries (2) but they are also hypothesized to contribute to the production of novel genetic combinations that help drive rapid diversification (9, 44, 82) and to act as bridges that facilitate adaptive introgression.

Viewing *Vitis* as a syngameon has conceptual and practical consequences. Conceptually, it adds a temperate, perennial plant clade to a growing list of systems where species boundaries are porous and hybridization is a central evolutionary force (44, 80). It also reframes adaptive repeatability. When adaptation is fueled primarily by introgression and standing variation, the evolutionary unit is not an isolated species but a shared, geographically structured pool of adaptive variants that circulates among species. Practically, it implies that adaptively important variation in wild *Vitis* is not neatly partitioned among species but distributed across the syngameon, with hybrid swarms and contact zones serving as potential reservoirs for rootstock improvement and climate-resilience (25, 83, 84). Given our observations, we envision three central challenges for future work: i) to determine whether the prominence of adaptive introgression in *Vitis* is a general property of plant radiations or specific to long-lived, interfertile genera with extensive sympatry, ii) to identify specific loci that are not porous and thus may maintain species’ boundaries over evolutionary time, and iii) to test whether the temporal decay of repeatability (Fig. 5*D*) generalizes to other syngameon-like systems.

## Materials and Methods

For full materials and methods, see *SI Appendix*.

Our dataset combined newly generated and previously published whole-genome resequencing data. All short-read data were adapter and quality trimmed, mapped to the *V. arizonica* v2.0 reference genome (32) and genotyped with the GATK4-based protocols (85–87). SNPs were pruned for linkage disequilibrium (LD) using PLINK (88, 89) and used for ancestry inference with NGSAdmix (K = 1 to 20), with the optimal K selected by the ΔK statistic (33). Species-level phylogenies were inferred from LD-pruned SNPs using RAxML (90) and SVDquartets in PAUP (91). K-mer distances were computed with MASH (92). Divergence times were estimated with BEAST2 (93). Introgression was quantified using Patterson’s D-statistic, the f4-ratio (94), the f-branch statistic (95), and TreeMix (96).

Species distribution models (SDMs) were generated for 14 North American *Vitis* species using BIOMOD2 (97) with three algorithms (Random Forest, Generalized Additive Models, MaxEnt), 10 bootstrap replicates, and bioclimatic variables from WorldClim2 (98). Occurrence data were obtained from Global Biodiversity Information Facility (GBIF) (99). The histories of four hybrid groups were investigated using ADMIXTURE, PCA, and triangular plots (triangulaR) (40). Hybridization timing was estimated for each individual using DATES (100). Local ancestry inference for chromosome painting was conducted using WinPCA (41).

Selective sweeps were identified using the Composite Likelihood Ratio (CLR) statistic implemented in SweeD (42). Species-specific demographic histories were inferred with *mushi* (101); neutral coalescent simulations were generated with msprime (102) under species-specific demographic models to establish significance thresholds. The evolutionary mechanism underlying each shared sweep was inferred using *rdmc* (43, 73). The relationship between shared sweep count and divergence time was assessed by Pearson correlation and PGLMM using MCMCglmm (103).

## Supplementary Material

Appendix 01 (PDF)

Dataset S01 (XLSX)

Dataset S02 (XLSX)

Dataset S03 (XLSX)

Dataset S04 (XLSX)

Dataset S05 (XLSX)

Dataset S06 (XLSX)

## Data Availability

Newly generated Illumina whole-genome sequencing reads for 114 accessions are deposited in the NCBI SRA under the BioProject accession PRJNA1424665 (104). Previously published whole-genome sequencing reads from 525 accessions can be accessed from the NCBI Sequence Read Archive (SRA) under the BioProject accessions PRJNA393611 (105), PRJNA842753 (32), PRJNA321480 (106), PRJNA984685 (38), and PRJCA009314 (39). Geographic occurrence data were obtained from the Global Biodiversity Information Facility (https://www.gbif.org/) (99). Environmental variables data are available from WorldClim (https://www.worldclim.org/) (98). Analysis pipelines and customized scripts used in this study are available via GitHub at https://github.com/wang-tianpeng/Vitis_PopGen (107).
