# Supplementary material for: The dynamics of introgression and parallel adaptation across North American Vitis species
Source: Proc Natl Acad Sci U S A. 2026 Aug 5;123(32):e2607754123. doi: 10.1073/pnas.2607754123 (PMC13462565; doi:10.1073/pnas.2607754123)
Supplement: Supplementary file 1 [file pnas.2607754123.sapp.pdf]

Supporting Information Text and Figures for

The dynamics of introgression and parallel adaptation across North American *Vitis* species

Tianpeng Wang[1*], Christopher J. Fiscus[1*], Jacob B. Landis[2], Noé Cochetel[3], Abraham Morales-Cruz[4], Dario Cantu[3,5], Jonas Aguirre-Liguori[6,&] and Brandon S. Gaut[1,&]

[1]Department of Ecology and Evolutionary Biology, University of California Irvine, Irvine, CA
[2] School of Integrative Plant Science, Section of Plant Biology and the L.H. Bailey Hortorium, Cornell University, Cornell, NY
[3] Department of Viticulture and Enology, University of California Davis, Davis, CA
[4] Joint Genome Institute, Lawrence Berkeley National Laboratory, Berkeley, CA
[5] Genome Center, University of California Davis, Davis, CA
[6] Departamento de Ecología Tropical, Campus de Ciencias Biológicas y Agropecuarias, Universidad Autónoma de Yucatán, Merida, Mexico
[*]co-first authors
[&]co-corresponding authors; Addresses for Correspondence
Brandon Gaut, bgaut@uci.edu
Jonas Aguirre-Liguori, jonas.aguirre@correo.uady.mx

This PDF file includes:
Supplementary Materials and Methods
Supplementary Figures 1 to 24

Supplementary Materials and Methods

Genome sequencing and SNP calling

Our dataset consists of sequencing reads from published work and newly generated sequences. The previously published data are available from the NCBI Short Read archive at PRJNA393611 (1), PRJNA842753 (2), PRJNA321480 (3), PRJNA984685 (4) and PRJCA009314 (5). New Illumina whole genome sequencing reads from 114 accessions were generated following the methods described in (6) and were deposited under PRJNA1424665. Detailed information about sources, species and accession sampling locations can be found in Table S1.

Sequencing reads were adapter and quality trimmed using Trimmomatic 0.39(7) with the following options: "ILLUMINACLIP:"$ADAPTERSPE":2:30:10 LEADING:3 TRAILING:3 SLIDINGWINDOW:4:20 MINLEN:60". The resulting reads were then mapped to haplotype 1 of the *V. arizonica* v.2.0 genome assembly (2) using bwa-mem 0.7.12-r1039(8) with the default options. Alignments were sorted and converted to binary format with samtools 1.10(9). Duplicates were marked with picard MarkDuplicates integrated into GATK 4.2.6.1(10–12). Samples were genotyped using GATK HaplotypeCaller in GVCF mode prior to joint calling with GenotypeGVCFs. Raw variants were filtered with bcftools 1.17(9) to retain only biallelic SNPs with site quality of 20 or better, that passed the GATK recommended hard filters (exclude "QD < 2 | FS > 60 | SOR > 3 | MQ < 40 | MQRankSum < -12.5 | ReadPosRankSum < -8.0"), had a MAF >= 0.01, and had site depth greater than the mean plus standard deviation of depth across all sites. Finally, we filtered for missing data by removing individuals with greater than 70% missing calls across sites and sites with greater than 5% calls missing across individuals.

LD pruning and admixture

SNPs were pruned for linkage disequilibrium using PLINK 2.0 (13, 14) with a pairwise sliding-window approach (50 SNP windows, 10 SNP step size) and an R^2 threshold of 0.20. The pruned SNP set was then filtered to remove variants with a minor allele frequency (MAF) < 0.05. PCA was performed in PLINK 2.0 using a variance-standardized relationship matrix. Admixture analysis on genotype likelihoods was performed with NGSAdmix v32 (15) considering K = 1 to 20 subpopulations at 20 replicates per K. Plink-formatted genotypes were converted to beagle format with ANGSD v0.940 (16), which was used as input to NGSAdmix. The optimal number of ancestral populations was determined by calculating the delta K statistic (17) (**Fig. S2**). Admixture data was then processed and visualized using the R package pophelper (18).

Phylogenetic inference

We constructed maximum likelihood (ML) phylogenetic trees using linkage disequilibrium (LD)-pruned SNP datasets from all samples. IQ-TREE v2.2.0 (19) was used to infer phylogenies under the best-fit nucleotide substitution model (GTR+R7), as identified by built-

in ModelFinder (20). Bootstrap values for each node were calculated using the ultrafast bootstrap method [UFboot2; (21)] with 1,000 replicates. The resulting trees were then visualized using the R package ggtree v4.0.4 (22). For all trees we designated *Leea guineensis* as the outgroup.

For species-level phylogenetic inference, we first removed samples potentially influenced by admixture using two complementary approaches. First, we screened for outliers within each species using principal component analysis. For species represented by three or more samples, we calculated pairwise Euclidean distances among individuals in multidimensional PC space and computed modified Z-scores based on each sample's median distance to others from the same species. Samples with a modified Z-score > 3.5 were flagged as putatively admixed. Second, we manually examined ancestry estimates from the admixture analysis to confirm PCA-identified outliers and to evaluate species represented by fewer than three samples. Samples flagged by either approach were considered putatively admixed and were not considered in subsequent analyses.

From the remaining samples, we randomly selected up to three individuals per species to represent intraspecific genetic diversity on the phylogeny. With this curated dataset, species relationships were verified using two approaches: a concatenated SNP tree inferred with RAxML-ng v1.1.0 (23) using the GTR+G model of molecular evolution with 100 bootstrap replicates and a coalescent-based inference using SVDquartets in Paup v4a166 (24) by evaluating all quartets and using 1000 bootstrap replicates. To estimate divergence times among species, we constructed a clock-calibrated phylogeny using BEAST2 v2.7.7 (25). We selected the GTR nucleotide substitution model with four gamma-distributed rate categories and applied a relaxed molecular clock. A Calibrated Yule prior was specified for tree branching processes with a fixed topology from SVDquartets. Markov Chain Monte Carlo (MCMC) sampling was performed for 10 million generations, with tree states sampled every 1,000 generations and discarding the initial 10% as burn-in. Fossil calibration priors were defined using a log-normal distribution based on previously published divergence estimates between subgenera *Vitis* and *Muscadania* (26). The posterior distribution of calibrated trees was visualized using DensiTree v2.6.4 (27).

To quantify site-level phylogenetic discordance, we calculated site concordance factors (sCFs) using IQ-TREE with the `--scfl 1000` parameter (19). The SVDquartets species tree was used as the reference topology, and sCFs were estimated from the genome-wide LD-pruned SNP matrix. For each internal branch, IQ-TREE sampled 1,000 quartets and estimated the proportion of decisive alignment sites supporting the reference-tree branch, as well as the proportions supporting the two alternative nearest-neighbor interchange topologies. The resulting sCF and sDF1 values were visualized on the reference tree as percentages.

To generate a reference-free phylogeny, K-mer–based sketches were generated for each sample from the trimmed sequencing reads using mash v2.3 with default parameters (28). Pairwise mash distances were computed using *mash dist*, yielding a distance matrix

summarizing genome-wide sequence similarity. This matrix was used to infer a neighbor-joining tree using the *nj* function in the R package ape v1.3 (29). The plastid genomes were assembled from trimmed sequencing reads using getOrganelle v.1.7.7.0 (30) with default parameters. For samples in which a complete plastid genome was not recovered, assemblies were reattempted using the parameters `-R 30 -w 95 –disentangle-time-limit 7200 –max-reads inf`, with the *Vitis vinifera* plastid genome (NCBI RefSeq NC_007957.1) used as a seed reference.

Resulting plastid assemblies were annotated with CHLOE (https://chloe.plastid.org/annotate.html) via its python API. Based on these annotations, coding sequences corresponding to the 101 plastid genes present in all 551 assemblies were extracted using bedtools v.2.31.1 getfasta (31). We also explored the use of non-coding plastid regions from complete plastome assemblies. As the complete plastome assemblies were available for only a limited number accessions, we retained the coding regions shared across all 551 assemblies to maximize taxon sampling and comparability across samples. Sequences for each gene were aligned independently using MAFFT v.7.505 (32) with the options `--maxiterate 2 –retree 2`. Individual gene alignments were concatenated into a supermatrix using seqkit (v2.4.0) concat (33) The concatenated alignments were then used to infer a maximum likelihood phylogeny using RAxML-ng v1.1.0 using the GTR+G model of molecular evolution and 100 bootstrap replicates. We calculated the Generalized Robinson-Foulds (GRF) distance between tree topologies using the R package TreeDist v.2.11.1 (34). This metric quantifies tree discordance on a normalized scale from 0 (identical topologies) to 1 (no shared information).

Detecting Introgression between Species

To investigate introgression between various *Vitis* species, we initially applied Patterson's D statistic (35) with a four-taxon topology using *M. rotundifolia* as the outgroup (O). We tested whether any of two taxa (P1 or P2) shared an excess of alleles with a potential introgression partner (P3). D-statistics for all possible combinations of species trios within met inferred phylogenetic relationships were computed using the Dtrios module in Dsuite v0.5 (36) using default parameters. Statistical significance and standard errors for D-values were obtained using block jackknifing for non-overlapping contiguous genomic blocks. We also calculated the overall f4-admixture ratio, which quantifies the proportion of introgressed genome segments from a donor lineage, utilizing the admixr package v0.10 with default parameters (37). We also calculated the f-branch statistic from Dsuite fbranch module with default parameters (36), which assigns migration events to specific internal branches on the phylogeny. We applied Treemix v1.3 (38) by constructing a phylogenetic tree without migration events using the LD-pruned SNP data and *M. rotundifolia* as the outgroup. We evaluated models by adding between 1 and 19 migration edges. The optimal number of migration events was determined using optM package v0.1.5 (39).

Species Distribution Modeling

We generated species distribution models (SDMs) for 14 *Vitis* species from North America, emphasizing present-day distributions and expanding on statistical approaches previously employed to predict future SDMs (40). Geographic occurrence data were obtained from the Global Biodiversity Information Facility (GBIF) (41). Current bioclimatic variables corresponding to occurrence data were downloaded from WorldClim2 (42), based on mean observations from 1970 to 2000. After obtaining occurrence and bioclimatic variables, we followed the methodology described in two previous publications (43, 44), with some modifications. Briefly, for each species we first cleaned the occurrences using the R package CoordinateCleaner v2.0 (45), to remove potential erroneous locations. Next, we used the *M_simulationR* function in the package Grinnell package v.0.0.22 (46), and the *mask* function in terra package (47) to delimit the accessible area (M Layer) for each species. The M layer was built based on simulated individual dispersal and the overlap between the remaining occurrence records and the world's terrestrial ecoregions (48). Additionally, for the bioclimatic variables, we used the *CorSelect* function of the fuzzySim package (49) to select uncorrelated variables (R < 0.8) based on a variance inflation factor.

The cleaned occurrences and the selected bioclimatic variables were used to build SDMs with the R package BIOMOD2 v. 4.2-4 (50). For each species, we ran 10 bootstrap replicates, using 70% and 30% of locations for training and testing with each of three algorithms (Random Forest, Generalized Additive Models and Maxent) to build 30 models per species. Finally, we generated ensemble models by weighting individual models according to their ROC (>0.8) and TSS (>0.6) values, producing final binarized and projected distributions. One species, *Vitis berlandieri,* had no entries in GBIF; we only were able to obtain records from the locations that were sampled for this study. We were therefore unable to run SDMs for this species but instead estimated its geographic distribution using a convex hull approach with the *hull* function in the terra v.1.8-70 package (47).

Geographic centroids, species overlaps and landscape-scale admixture analyses

Once binarized SDMs were obtained, we estimated the geographic centroid of each species using the *centroid* function in the terra package (47). For each pixel in the species distribution *SpatRaster*, we extracted the geographic coordinates and calculated the Euclidean distance from each pixel to the species' geographic centroid. The values were then rasterized to produce a *SpatRaster* representing the distance of each location to the geographic centroid. We exclude the putative hybrid species (*V.* × *doaniana* and *V.* × *champinii*) pairs to avoid confounding species-level patterns. We also analyzed the geographic overlap and distance between geographic centroids for a total of 105 possible pairs of species with SDMs available. For each species pair we identified the area where the species overlapped and used the *expanse* function in the terra package to obtain the overlap area in Km^2. Species pairs were categorically defined as i) overlapping North American species (SDM overlap > 0 km^2);ii) non-overlapping North American species; iii) species

contrasts that included Eurasian taxa (*V. vinifera*, *V. sylvestris*, and *V. amurensis*). We also estimated the geographic distance between the centroids ($d_{centroid}$) of each pair of species, using the *distGeo* function in the Geosphere v.1.5-18 package (51). We compared the maximum D and pairwise matrices using Multiple Regression Models, as implemented in the *MRM* function in the Ecodist package (52). Linear models were calculated in R.

To assess the relationship between admixture proportions of individuals and their geographic location, we calculated metrics that summarize the admixture proportion for each individual. We used two: 1) the maximum proportion from a single species (maxQ) based on population structure at K = 13; and 2) the effective number of contributing genomes [effN = exp(H), where H is the Shannon entropy estimated as $H = - \sum_{i=1}^{K} q_i \log (q_i)$]. We report results based on maxQ, but results with effN were qualitatively identical. For the 233 individuals with available sampling locations (**Table S4**), we calculated the Euclidean distance to their geographic centroids using distGeo (51). We estimated the association between the admixture metrics and their distance to the geographic centroid using linear models as implemented with the lm function in R.

Investigating the History of Putative Hybrid Species

To investigate the history of hybrid groups, we assumed the parentage of each group from admixture results and/or from the existing literature [e.g, (53)] (**Fig. 3 and Fig. S14**). For each of four hybrid groups (**Table S4**), we generated eight *in silico* F1 hybrids as a control group by combining SNP variants from randomly chosen six individuals of each assumed parental species. We performed the same analyses on each of the four sample groups. The first was PCA and admixture analyses, following the procedures described above. We also used the HIest method implemented in triangulaR (54) , which explicitly assesses hybrid status by estimating parental individuals, backcrosses, F1, and later hybrid generations (≥F2) based on ancestry (S) and heterozygosity (H) at diagnostic loci (55). Ancestry-informative markers were selected by identifying loci with allele frequencies ≥0.9 in one parental species and ≤0.1 in the other.

The timing of hybridization events for each hybrid individual were estimated using DATES v753 (Distribution of Ancestry Tracts of Evolutionary Signals) (56). DATES infers the time of population admixture by converting the average length of ancestry blocks into generations, based on a recombination rate of 6.4e-8 per base pair per generation (57). The analysis was performed using the following parameters: binsize = 0.00005, maxdis = 1.0, runmode = 1, chithresh = 0, mincount = 1, qbin = 50, lovalfit = 0.1, and runfit, afffit, and zdipcorrmode set to YES. We inferred local ancestry across the genome for each hybrid-parent group using the window-based PCA, WinPCA v1.2 (58), which performed PCA analyses within sliding genomic windows (200 Kb window 10 Kb step) across all 19 chromosomes. We used ggplot2 to generate chromosome painting diagrams.

Demography, selective sweep identification and population genetic simulations

We studied population genetic properties, including demography and selective sweeps, of eight *Vitis* species with n > 15 individuals **(Table S4)**. We used a three-tiered approach to detect selective sweeps. First, we inferred the demographic history of each species. We used mushi v0.2 (59) to estimate changes in effective population size (*Ne*) over time. The unfolded site frequency spectrum (SFS) was inferred for ten randomly selected individuals per species using est-sfs (60), with ancestral states polarized using *M. rotundifolia* as an outgroup. To prevent overfitting with limited sample sizes, regularization parameters were set to ridge_penalty = 1e4, alpha_spline = 1e4, and trend_penalty = 1e2. Analyses assumed a mutation rate of 5.4e-9 per nucleotide per year (61) and a generation time of three years (62).

Second, we generated neutral replicates for each species using coalescent simulations msprime (63) under species-specific demographic models. For each species, we set simulation parameters using effective population size trajectories inferred from mushi, incorporating ancestral population sizes, bottleneck population size and timing, and exponential growth where applicable. Simulations assumed a mutation rate of 5.4e-9 per nucleotide per year and a recombination rate of 6.4e-8 per base pair per generation (57). For each species, we generated 1000 independent neutral genomic regions matching the length and SNP density of our empirical dataset. We applied SweeD v4.0 (64) to each simulated dataset, using the same parameters as for the empirical data, to generate a distribution of maximum CLR values across 1,000 simulations, establishing an empirical null distribution.

Finally, we used SweeD to identify genomic regions under recent positive selection, using grid sizes of 10 kb and default parameters. To define coherent selective sweep regions from CLR peaks, we fitted cubic smoothing splines to the CLR scores along each chromosome using the *smooth.spline* function in R. Peaks exceeding 99.5% of the null distribution were considered significant. Outlier regions exceeding the significance threshold were merged when they were within 50 kb of each other (65).

Comparison of selective sweeps among different species

After identifying sweep regions for each species, we performed pairwise comparisons to quantify shared sweeps among species. A sweep region was defined as shared if the overlapping genomic segment constituted >60% of the total region size in at least one of the species in the pair. To test whether the observed amount of sharing was statistically significant, we performed a permutation test. For each species, we generated a null distribution by creating 1,000 replicates of randomly sampled genomic windows that matched the number and size distribution of the observed sweeps. These random regions were sampled from non-overlapping portions of the genome to avoid bias from clustered sweep signals. For each permutation replicate, we calculated the total size of the overlap between these random windows from one species and the observed sweep regions from the other species. The statistical significance of the observed overlap was then assessed by calculating an empirical P-value, defined as the proportion of the 1,000 null replicates that yielded an overlap size greater than the observed overlap. Finally, to characterize the potential biological

functions associated with signatures of selection, we performed Gene Ontology (GO) enrichment analysis on genes located within the identified selective sweep regions using the R package clusterProfiler v4.18 (66).

Inferring modes of convergent adaptation

For sweep regions identified as shared between species pairs, we used rdmc (67, 68) to infer the most likely mode of convergent adaptation. This method compares the composite likelihoods of four distinct evolutionary scenarios: 1) neutral evolution, 2) independent selective sweeps acting on different mutations, 3) selection on shared standing genetic variation present in the ancestral population. 4) adaptive introgression (migration) of beneficial alleles between species. We followed the model configuration and parameterization settings adopted from previous study (65). Before model fitting, we expanded each identified sweep region by adding 10% of its total length to ensure sufficient flanking sequence for accurately estimating patterns of haplotype decay and recombination. Due to substantial differences in sweep region sizes across species pairs, we normalized SNP density by subsetting the total number of sites within each, maintaining a density between 1,000 and 100,000 SNPs per region. The recombination rate for each sweep region was approximated based on the genome-wide genetic map, assuming a recombination rate of 6.4e-8 (57). Each shared sweep region was assigned to the evolutionary model with the highest log-composite likelihood.

To assess confidence in model assignments, we calculated the difference between the highest log-likelihood and the neutral model log-likelihood; regions with ΔCLE > 2.0 were considered to have support for a specific non-neutral evolutionary model. Comparisons between species divergence time and the number of shared sweeps were investigated with Pearson correlation coefficients. To account for the non-independence of species pairs due to shared evolutionary history, correlations were calculated using the Phylogenetic Generalized Linear Mixed Model (PGLMM) from MCMCglmm v2.36 package (69).

CITATIONS to SUPPLEMENTAL TEXT

1. Z. Liang, *et al.*, Whole-genome resequencing of 472 Vitis accessions for grapevine diversity and demographic history analyses. *Nat. Commun.* **10**, 1190 (2019).
2. A. Morales-Cruz, *et al.*, Multigenic resistance to Xylella fastidiosa in wild grapes (Vitis sps.) and its implications within a changing climate. *Commun Biol* **6**, 580 (2023).
3. G. Magris, *et al.*, The genomes of 204 Vitis vinifera accessions reveal the origin of European wine grapes. *Nat. Commun.* **12**, 7240 (2021).
4. N. Cochetel, *et al.*, A super-pangenome of the North American wild grape species. *Genome Biol.* **24**, 290 (2023).
5. Y. Dong, *et al.*, Dual domestications and origin of traits in grapevine evolution. *Science* **379**, 892–901 (2023).
6. A. Morales-Cruz, *et al.*, Introgression among North American wild grapes (Vitis) fuels biotic and abiotic adaptation. *Genome Biol.* **22**, 254 (2021).
7. A. M. Bolger, M. Lohse, B. Usadel, Trimmomatic: a flexible trimmer for Illumina sequence data. *Bioinformatics* **30**, 2114–2120 (2014).
8. H. Li, Aligning sequence reads, clone sequences and assembly contigs with BWA-MEM. *arXiv preprint*. 1303.3997 (2013).
9. P. Danecek, *et al.*, Twelve years of SAMtools and BCFtools. *Gigascience* **10**, 2 (2021).
10. A. McKenna, *et al.*, The Genome Analysis Toolkit: a MapReduce framework for analyzing next-generation DNA sequencing data. *Genome Res.* **20**, 1297–1303 (2010).
11. M. A. DePristo, *et al.*, A framework for variation discovery and genotyping using next-generation DNA sequencing data. *Nat. Genet.* **43**, 491–498 (2011).
12. R. Poplin, *et al.*, Scaling accurate genetic variant discovery to tens of thousands of samples. *bioRxiv* 201178 (2018).
13. S. Purcell, *et al.*, PLINK: a tool set for whole-genome association and population-based linkage analyses. *Am. J. Hum. Genet.* **81**, 559–575 (2007).
14. C. C. Chang, *et al.*, Second-generation PLINK: rising to the challenge of larger and richer datasets. *Gigascience* **4**, 7 (2015).
15. L. Skotte, T. S. Korneliussen, A. Albrechtsen, Estimating individual admixture proportions from next generation sequencing data. *Genetics* **195**, 693–702 (2013).
16. T. S. Korneliussen, A. Albrechtsen, R. Nielsen, ANGSD: Analysis of Next Generation Sequencing Data. *BMC Bioinformatics* **15**, 356 (2014).
17. G. Evanno, S. Regnaut, J. Goudet, Detecting the number of clusters of individuals using the software STRUCTURE: a simulation study. *Mol. Ecol.* **14**, 2611–2620 (2005).
18. R. M. Francis, pophelper: An R package and web app to analyse and visualise population structure. *Mol. Ecol. Resour*. **17**, 27-32 (2017).
19. B. Q. Minh, H. A. Schmidt, O. Chernomor, D. Schrempf, M. D. Woodhams, A. von Haeseler, R. Lanfear, IQ-TREE 2: New Models and Efficient Methods for Phylogenetic Inference in the Genomic Era, *Mol. Biol. Evol.* **37**, 1530–1534 (2020).
20. S. Kalyaanamoorthy, B. Q. Minh, T. K. F. Wong, A. von Haeseler, L. S. Jermiin, ModelFinder: fast model selection for accurate phylogenetic estimates. *Nat. Methods* **14**, 587–589 (2017).
21. D. T. Hoang, O. Chernomor, A. von Haeseler, B. Q. Minh, L. S. Vinh, UFBoot2: Improving the ultrafast bootstrap approximation. *Mol. Biol. Evol.* **35**, 518–522 (2018).
22. G. Yu, Using ggtree to Visualize Data on Tree-Like Structures. *Curr. Protoc. Bioinformatics* **69**, e96 (2020).
23. A. M. Kozlov, D. Darriba, T. Flouri, B. Morel, A. Stamatakis, RAxML-NG: a fast, scalable and user-friendly tool for maximum likelihood phylogenetic inference. *Bioinformatics* **35**, 4453–4455 (2019).
24. D. L. Swofford, *PAUP* Phylogenetic Analysis Using Parsimony (and Other Methods)*. In Dictionary of Bioinformatics and Computational Biology (2002).
25. R. Bouckaert, *et al.*, BEAST 2: a software platform for Bayesian evolutionary analysis. *PLoS Comput. Biol.* **10**, e1003537 (2014).
26. Y. Wan, *et al.*, A phylogenetic analysis of the grape genus (Vitis L.) reveals broad reticulation and concurrent diversification during neogene and quaternary climate change. *BMC Evol. Biol.* **13**, 141 (2013).
27. R. R. Bouckaert, DensiTree: making sense of sets of phylogenetic trees. *Bioinformatics* **26**, 1372–1373 (2010).

28. B. D. Ondov, *et al.*, Mash: fast genome and metagenome distance estimation using MinHash. *Genome Biol.* **17** (2016).
29. E. Paradis, K. Schliep, ape 5.0: an environment for modern phylogenetics and evolutionary analyses in R. *Bioinformatics* **35**, 526–528 (2019).
30. J.-J. Jin, *et al.*, GetOrganelle: a fast and versatile toolkit for accurate de novo assembly of organelle genomes. *Genome Biol.* **21**, 241 (2020).
31. A. R. Quinlan, I. M. Hall, BEDTools: a flexible suite of utilities for comparing genomic features. *Bioinformatics* **26**, 841–842 (2010).
32. K. Katoh, D. M. Standley, MAFFT multiple sequence alignment software version 7: improvements in performance and usability. *Mol. Biol. Evol.* **30**, 772–780 (2013).
33. W. Shen, B. Sipos, L. Zhao, SeqKit2: A Swiss army knife for sequence and alignment processing. *Imeta* **3**, e191 (2024).
34. M. R. Smith, Information theoretic generalized Robinson-Foulds metrics for comparing phylogenetic trees. *Bioinformatics* **37**, 2077–2078 (2021).
35. E. Y. Durand, N. Patterson, D. Reich, M. Slatkin, Testing for ancient admixture between closely related populations. *Mol. Biol. Evol.* **28**, 2239–2252 (2011).
36. M. Malinsky, M. Matschiner, H. Svardal, Dsuite - Fast D-statistics and related admixture evidence from VCF files. *Mol. Ecol. Resour.* **21**, 584–595 (2021).
37. M. Petr, B. Vernot, J. Kelso, admixr—R package for reproducible analyses using ADMIXTOOLS. *Bioinformatics* **35**, 3194–3195 (2019).
38. J. K. Pickrell, J. K. Pritchard, Inference of population splits and mixtures from genome-wide allele frequency data. *PLoS Genet.* **8**, e1002967 (2012).
39. R. R. Fitak, OptM: estimating the optimal number of migration edges on population trees using Treemix. *Biol Methods Protoc* **6**, bpab017 (2021).
40. M. Zhang, *et al.*, The dynamics of wild Vitis species in response to climate change facilitate the breeding of grapevine and its rootstocks with climate resilience. *Hortic. Res.* **12**, uhaf104 (2025).
41. G. O. User, Occurrence Download. The Global Biodiversity Information Facility. https://doi.org/10.15468/DL.RXGDN7. Deposited 20 May 2025.
42. S. E. Fick, R. J. Hijmans, WorldClim 2: new 1-km spatial resolution climate surfaces for global land areas. *Int. J. Climatol.* **37**, 4302–4315 (2017).
43. J. A. Aguirre-Liguori, S. Ramirez-Barahona, B. S. Gaut, The evolutionary genomics of species' responses to climate change. *Nat. Ecol. Evol.* **5**, 1350–1360 (2021).
44. C. J. Fiscus, J. A. Aguirre-Liguori, G. R. J. Gaut, B. S. Gaut, Mutational load and adaptive variation are shaped by climate and species range dynamics in Vitis arizonica. *New Phytol.* **247**, 998–1014 (2025).
45. A. Zizka, *et al.*, CoordinateCleaner : Standardized cleaning of occurrence records from biological collection databases. *Methods Ecol. Evol.* **10**, 744–751 (2019).
46. F. Machado-Stredel, M. E. Cobos, A. T. Peterson, A simulation-based method for selecting calibration areas for ecological niche models and species distribution models. *Front. Biogeogr.* **13** (2021).
47. R. Hijmans, R. Bivand, E. Pebesma, M. Sumner, terra: Spatial data analysis (R Package version 1.8–0). https://github.com/rspatial/terra (2026).
48. D. M. Olson, *et al.*, Terrestrial ecoregions of the world: A new map of life on earth. *Bioscience* **51**, 933 (2001).
49. A. M. Barbosa, fuzzySim: applying fuzzy logic to binary similarity indices in ecology. *Methods Ecol. Evol.* **6**, 853–858 (2015).
50. W. Thuiller, B. Lafourcade, R. Engler, M. B. Araújo, BIOMOD – a platform for ensemble forecasting of species distributions. *Ecography (Cop.)* **32**, 369–373 (2009).
51. R. J. Hijmans, E. Williams, C. Vennes, Geosphere: spherical trigonometry. *R package version* **1** (2019).
52. S. Goslee, The ecodist package for dissimilarity-based analysis of ecological data. *R Coding for Ecology*, (Springer Nature Switzerland, 2026), pp. 99–122.
53. C. C. Heinitz, J. Uretsky, J. C. Dodson Peterson, K. G. Huerta-Acosta, M. A. Walker, "Crop Wild Relatives of Grape (Vitis vinifera L.) Throughout North America" in *North American Crop Wild Relatives, Volume 2: Important Species*, S. L. Greene, K. A. Williams, C. K. Khoury, M. B. Kantar, L. F. Marek, Eds. (Springer International Publishing, 2019), pp. 329–351.
54. B. J. Wiens, L. H. DeCicco, J. P. Colella, triangulaR: an R package for identifying AIMs and building triangle plots using SNP data from hybrid zones. *Heredity (Edinb.)* **134**,

251–262 (2025).
55. B. J. Wiens, J. P. Colella, That's Not a Hybrid: How to Distinguish Patterns of Admixture and Isolation By Distance. *Mol. Ecol. Resour.* **25**, e14039 (2025).
56. M. Chintalapati, N. Patterson, P. Moorjani, The spatiotemporal patterns of major human admixture events during the European Holocene. *Elife* **11**, e77625 (2022).
57. J. Zhu, *et al.*, Construction of a highly saturated Genetic Map for Vitis by Next-generation Restriction Site-associated DNA Sequencing. *BMC Plant Biol.* **18**, 347 (2018).
58. L. M. Blumer, J. M. Good, R. Durbin, WinPCA: a package for windowed principal component analysis. *Bioinformatics* **41**, btaf529 (2025).
59. W. S. DeWitt, K. D. Harris, A. P. Ragsdale, K. Harris, Nonparametric coalescent inference of mutation spectrum history and demography. *Proc. Natl. Acad. Sci. U. S. A.* **118**, e2013798118 (2021).
60. P. D. Keightley, B. C. Jackson, Inferring the probability of the derived vs. The ancestral Allelic state at a polymorphic site. *Genetics* **209**, 897–906 (2018).
61. Z. Liang, *et al.*, Whole-genome resequencing of 472 Vitis accessions for grapevine diversity and demographic history analyses. *Nat. Commun*. **10**, 1190 (2019).
62. Y. Zhou, M. Massonnet, J. S. Sanjak, D. Cantu, B. S. Gaut, Evolutionary genomics of grape (Vitis vinifera ssp. vinifera) domestication. *Proc. Natl. Acad. Sci. U. S. A.* **114**, 11715–11720 (2017).
63. F. Baumdicker, *et al.*, Efficient ancestry and mutation simulation with msprime 1.0. *Genetics* **220**, iyab229 (2022).
64. P. Pavlidis, D. Živković, A. Stamatakis, N. Alachiotis, SweeD: Likelihood-Based Detection of Selective Sweeps in Thousands of Genomes. *Mol. Biol. Evol.* 30, 2224-2234 (2013)
65. S. Tittes, *et al.*, The population genetics of convergent adaptation in maize and teosinte is not locally restricted. *eLife.* **12**, RP92405 (2025).
66. T. Wu, *et al.*, clusterProfiler 4.0: A universal enrichment tool for interpreting omics data. *The Innovation* **2**, 100141 (2021).
67. K. M. Lee, G. Coop, Distinguishing among modes of convergent adaptation using population genomic data. *Genetics* **207**, 1591–1619 (2017).
68. S. Tittes, Rdmc: An open source R package implementing convergent adaptation models of Lee and Coop (2017). *G3 (Bethesda)* **10**, 3041–3046 (2020).
69. J. D. Hadfield, MCMC methods for multi-response generalized linear mixed models: TheMCMCglmmRPackage. *J. Stat. Softw.* **33** (2010).

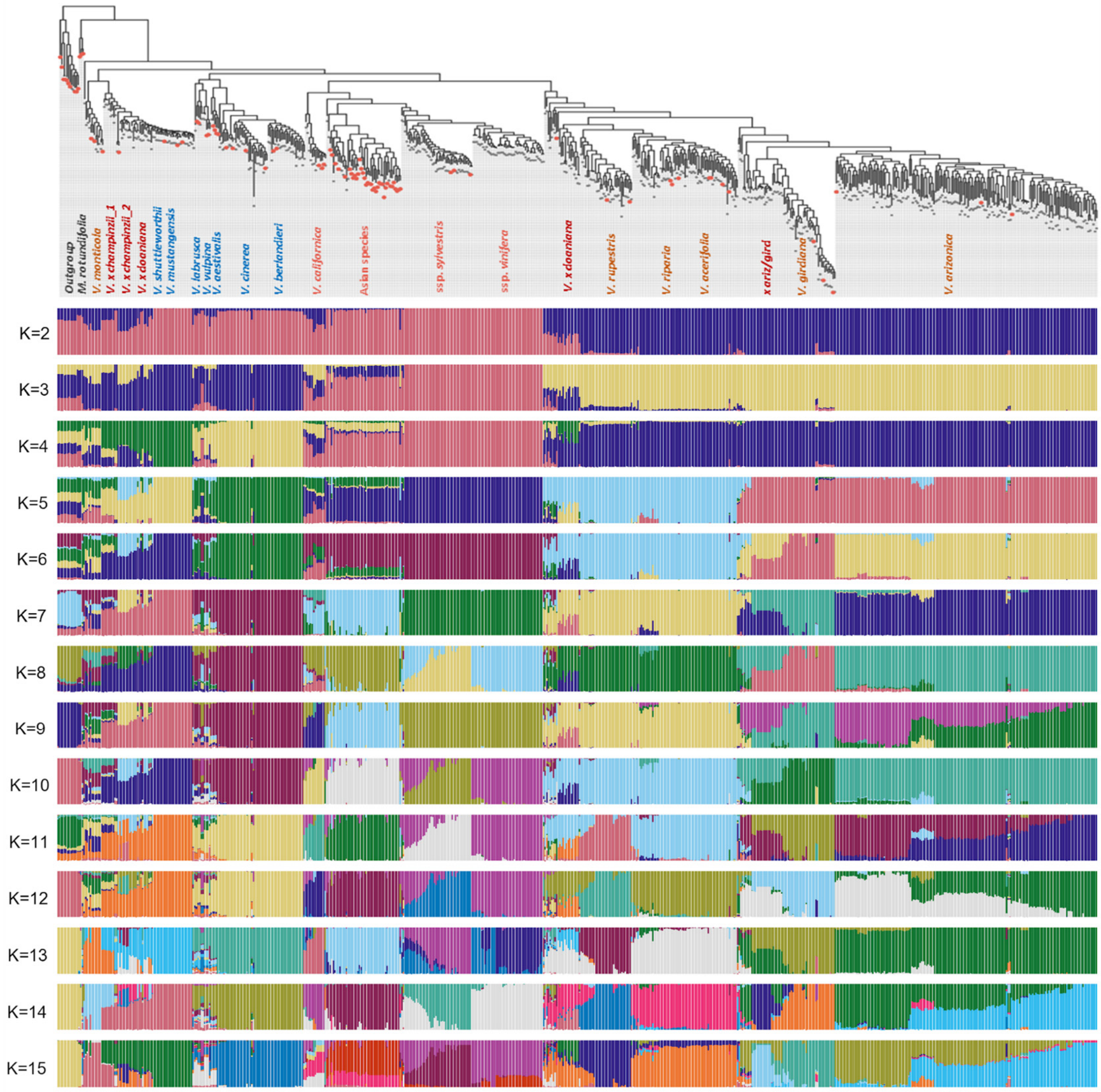

Supplementary Fig. 1. Phylogeny and population structure of Vitis species. (A) Maximum Likelihood tree of 639 Vitis accessions. Branch colors denote species. **(B)** Model-based Bayesian clustering performed with the number of ancestry kinship (K) from 2 to 15. The different colors of vertical bars represent contributions to the K-groups. Individuals are ordered based on their phylogenetic position.

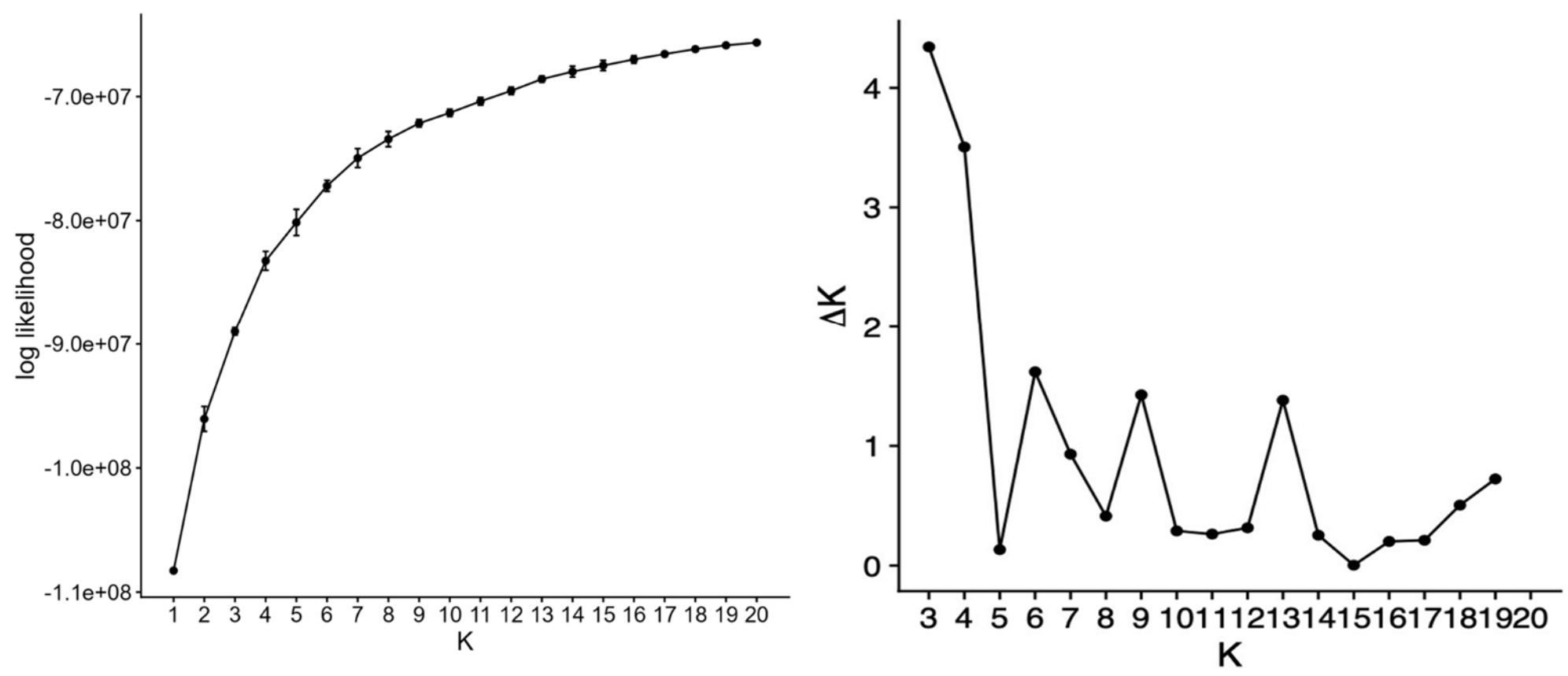

Supplementary Fig. 2. Log-likelihood value for different population structure K values. Cross-validation errors for each deltaK from 3 to 19 are shown in the right panel.

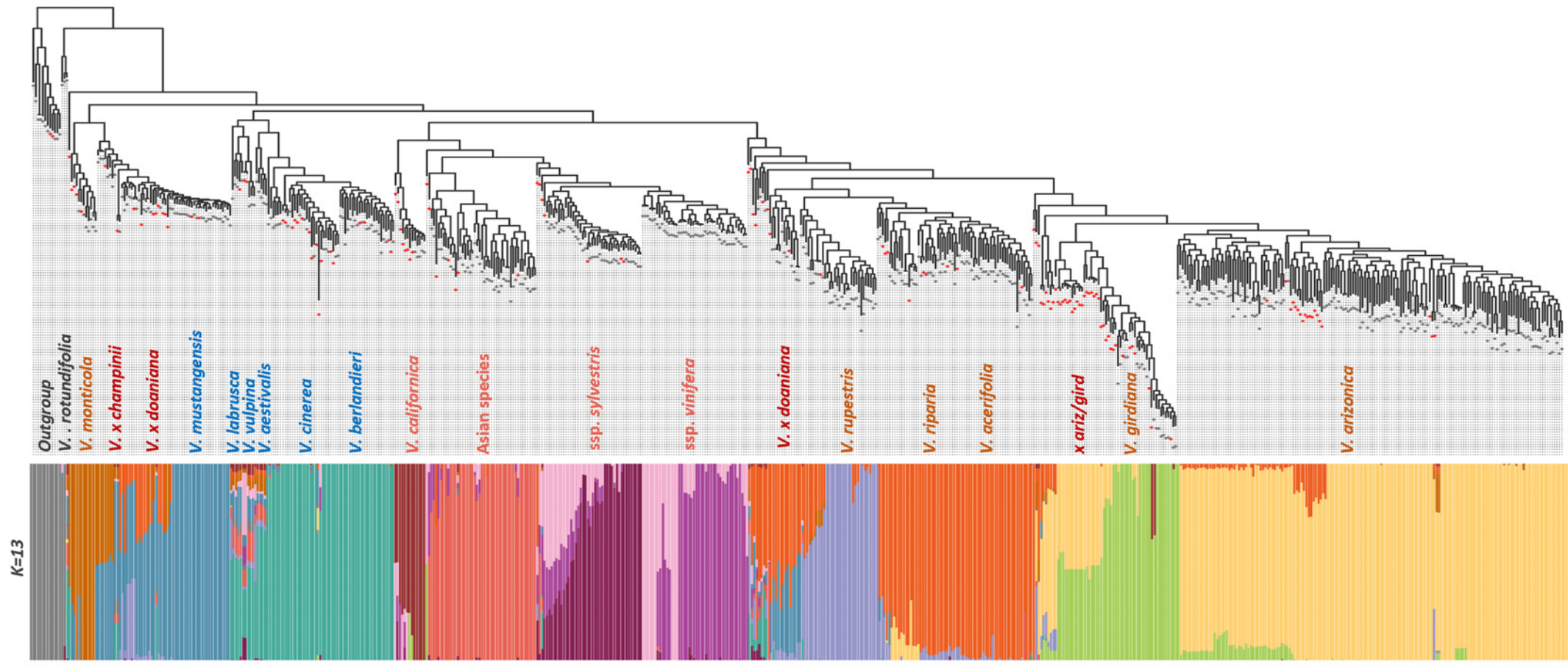

Supplementary Fig. 3. Identification of putatively mislabeled or highly admixed individuals. Individuals exhibiting substantial admixture (>20% ancestry from multiple clusters) or phylogenetic placement inconsistent with their assigned species were identified based on combined ancestry and phylogenetic analyses. Filtered individuals are denoted by red tips. Admixture profiles are shown in the K=13 bar plot below. Detailed information for all excluded individuals is provided in Table S2.

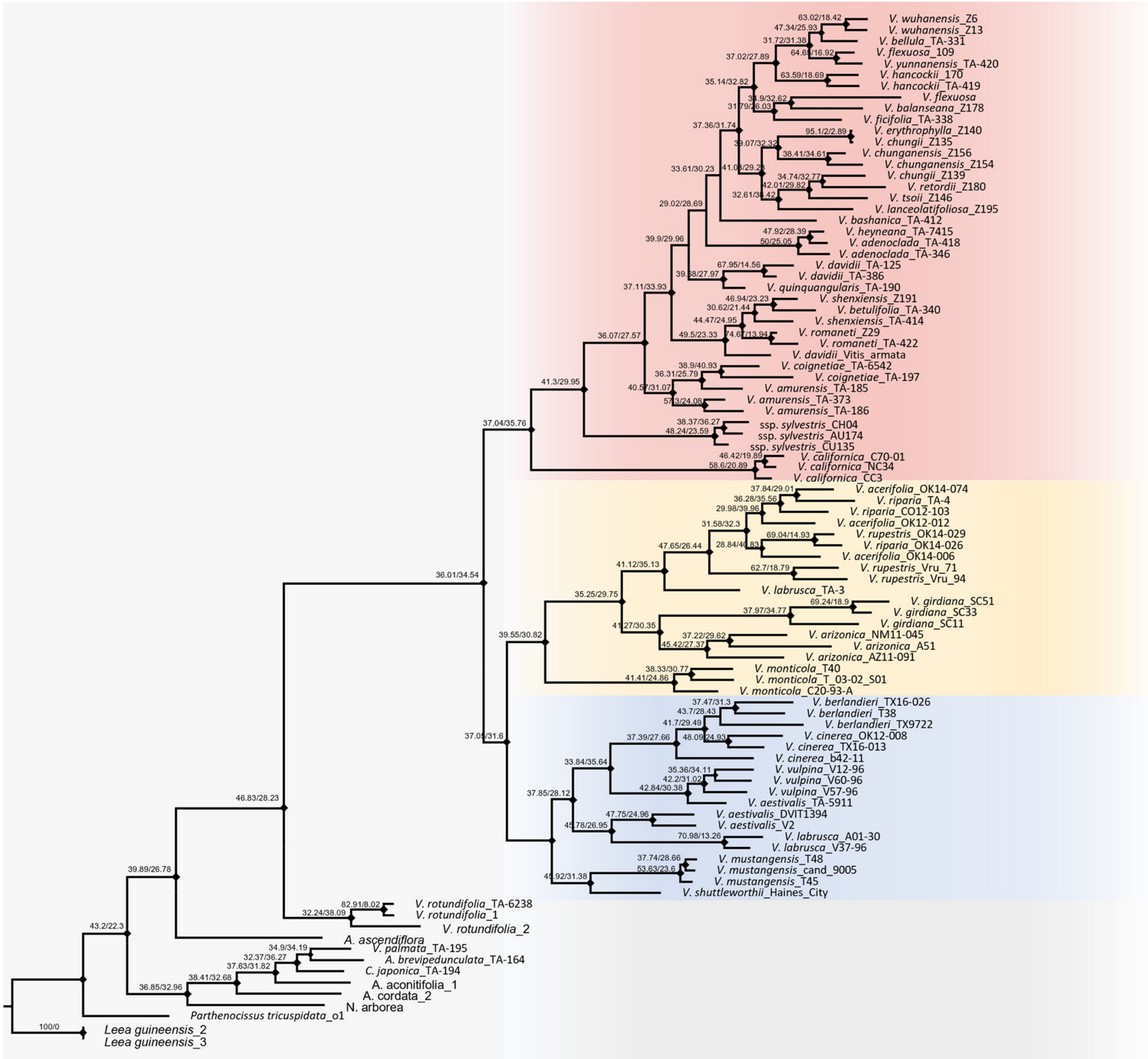

Supplementary Fig. 4. Quantification of phylogenetic concordance across the *Vitis* radiation using site concordance factors (sCF). The species-tree topology inferred with SVDQuartets is annotated with site concordance metrics calculated from the genome-wide LD-pruned SNP matrix. Numbers at internal nodes represent the site concordance factor and the first site discordance factor, respectively, expressed as percentages (sCF/sDF1). The sCF represents the proportion of decisive alignment sites supporting the focal branch in the reference topology, whereas sDF1 represents the proportion supporting the most frequent alternative topological arrangement. Background colors indicate major phylogenetic groups within *Vitis*.

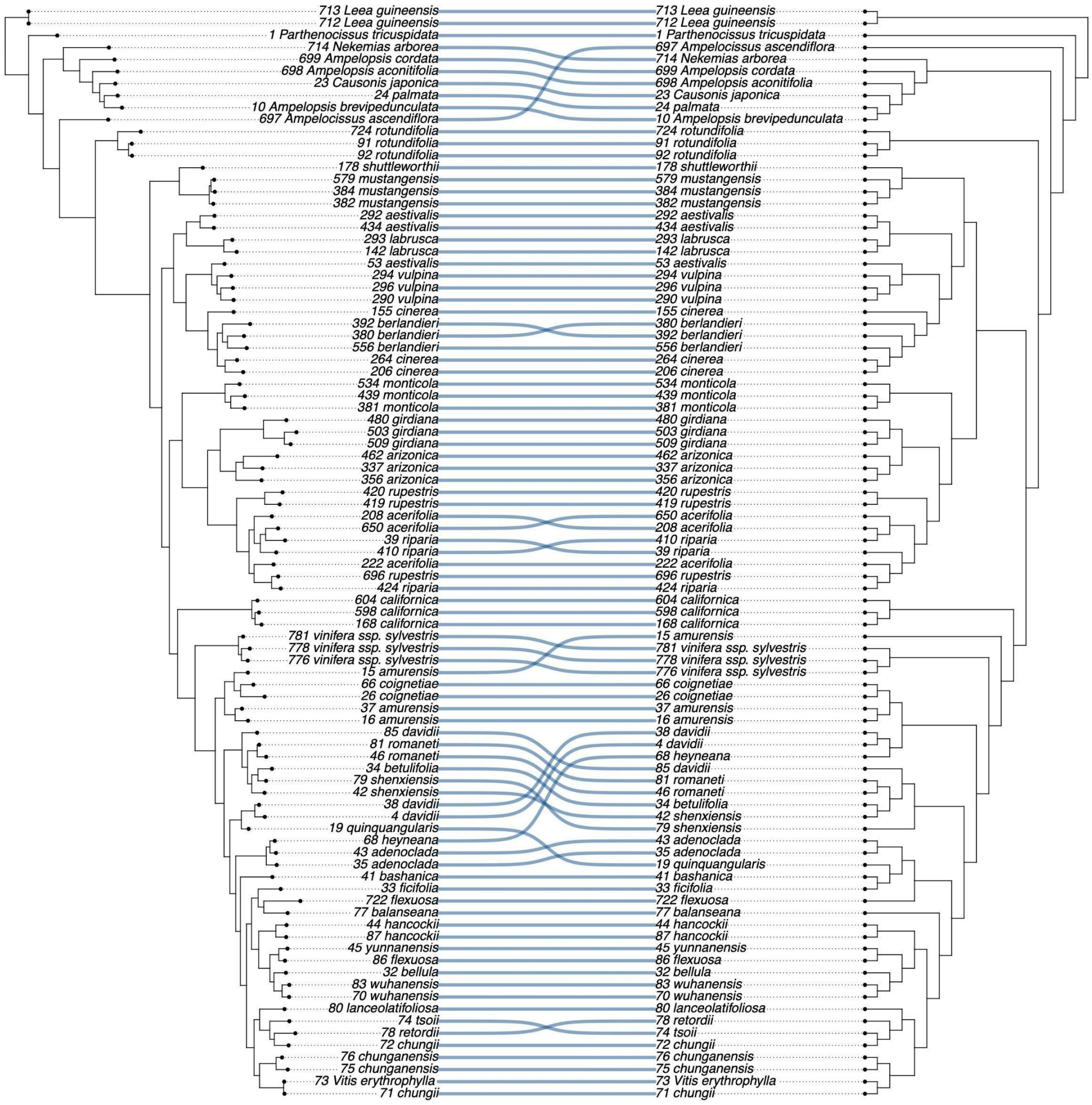

Supplementary Fig. 5. Comparison of species phylogenetic topologies inferred by Raxml and SVD tree. The tree on the left was inferred using RAxML (Maximum Likelihood), and the tree on the right was inferred using SVDquartets. Connecting lines link identical taxa between the two phylogenies to visualize topological concordance.

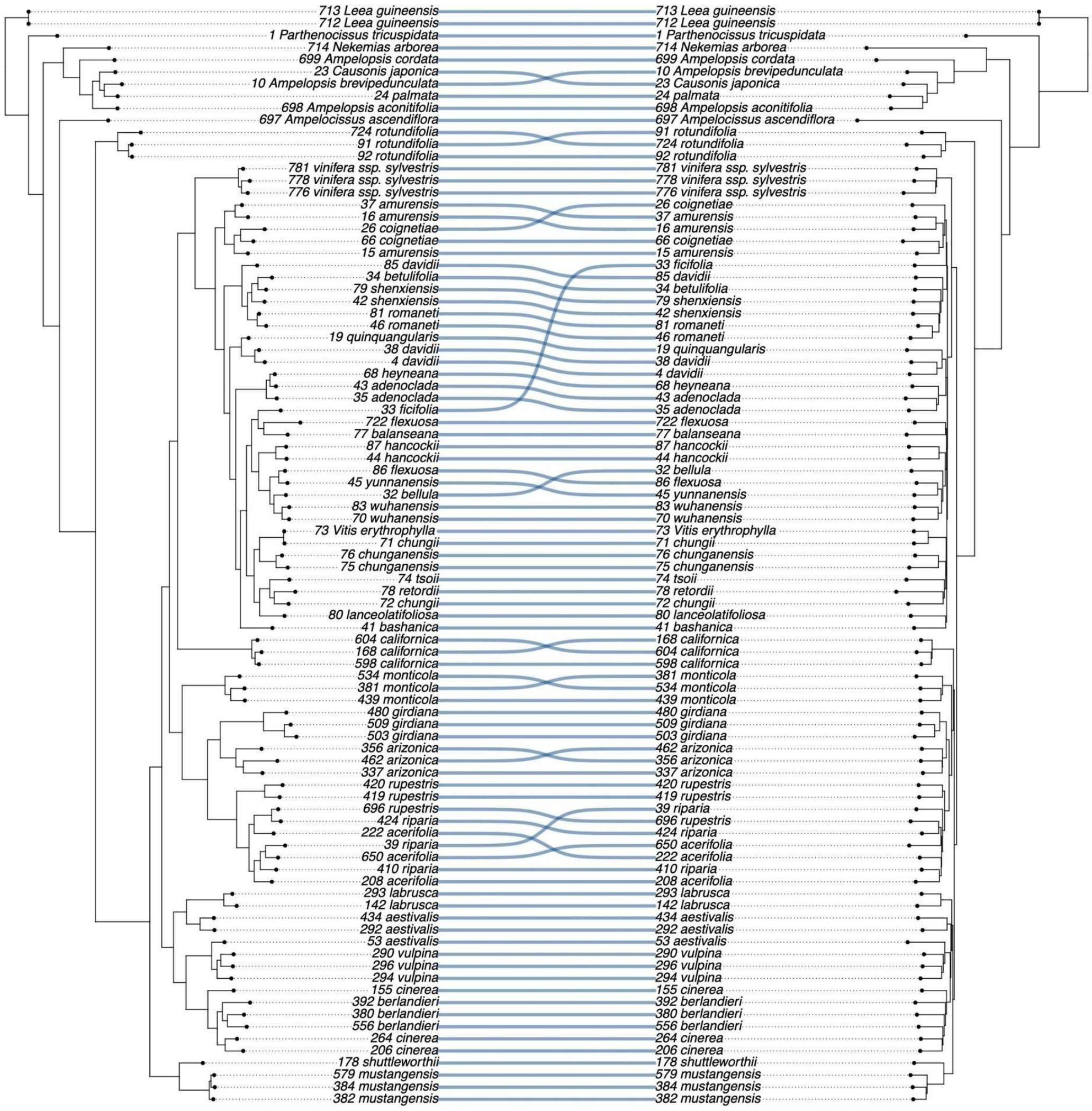

Supplementary Fig. 6. Comparison of Raxml and kmer tree. The figure displays the SNP-based RAxML tree on the left and the alignment-free K-mer tree on the right.

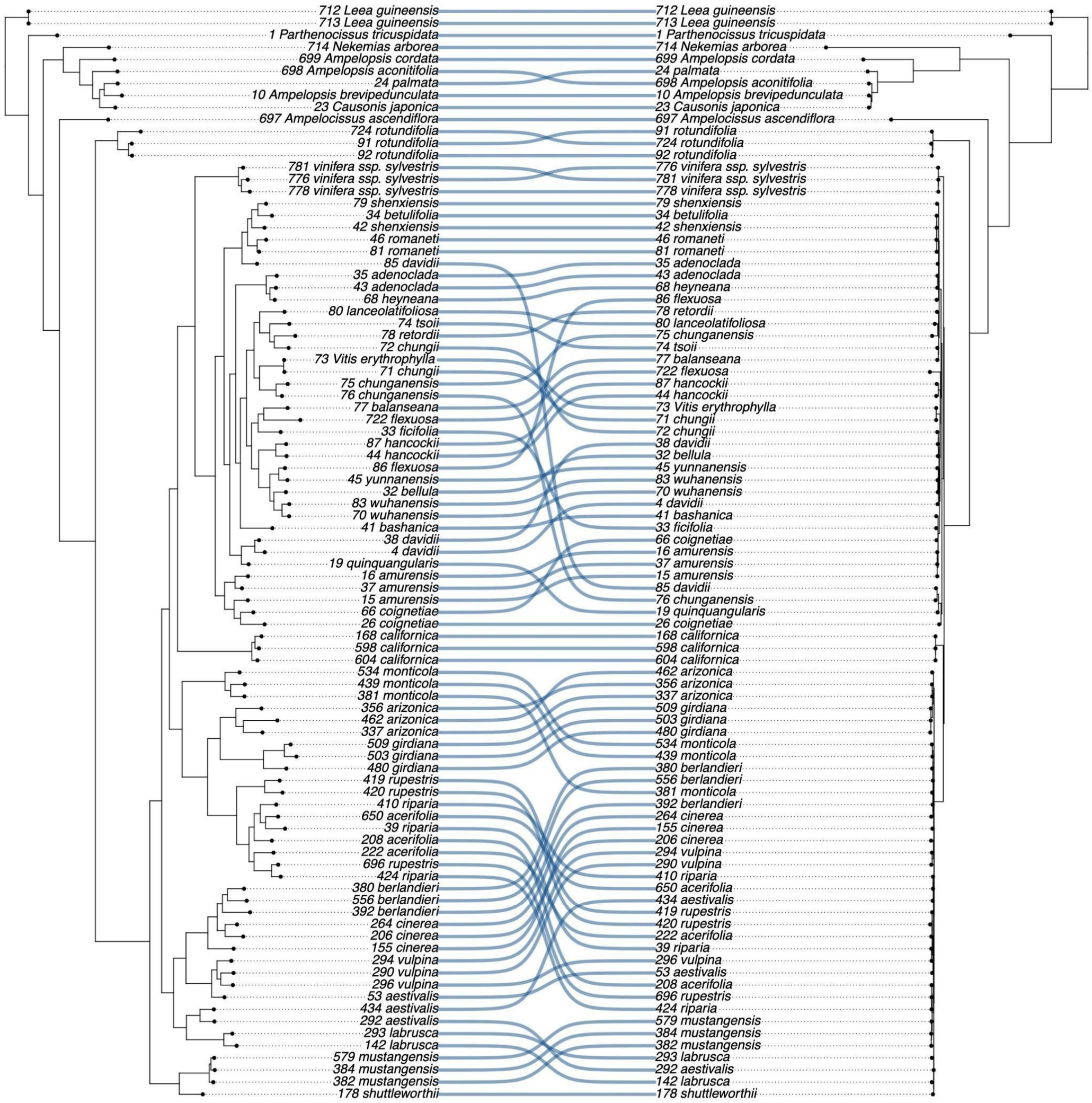

Supplementary Fig. 7. Comparison of nuclear RAxML and plastid (Pt) tree. The cophyloplot contrasts the nuclear SNP-based Maximum Likelihood tree (left) with the plastid haplotype-based tree (right). Connecting lines link corresponding taxa between the two phylogenies to illustrate differences in tree topology.

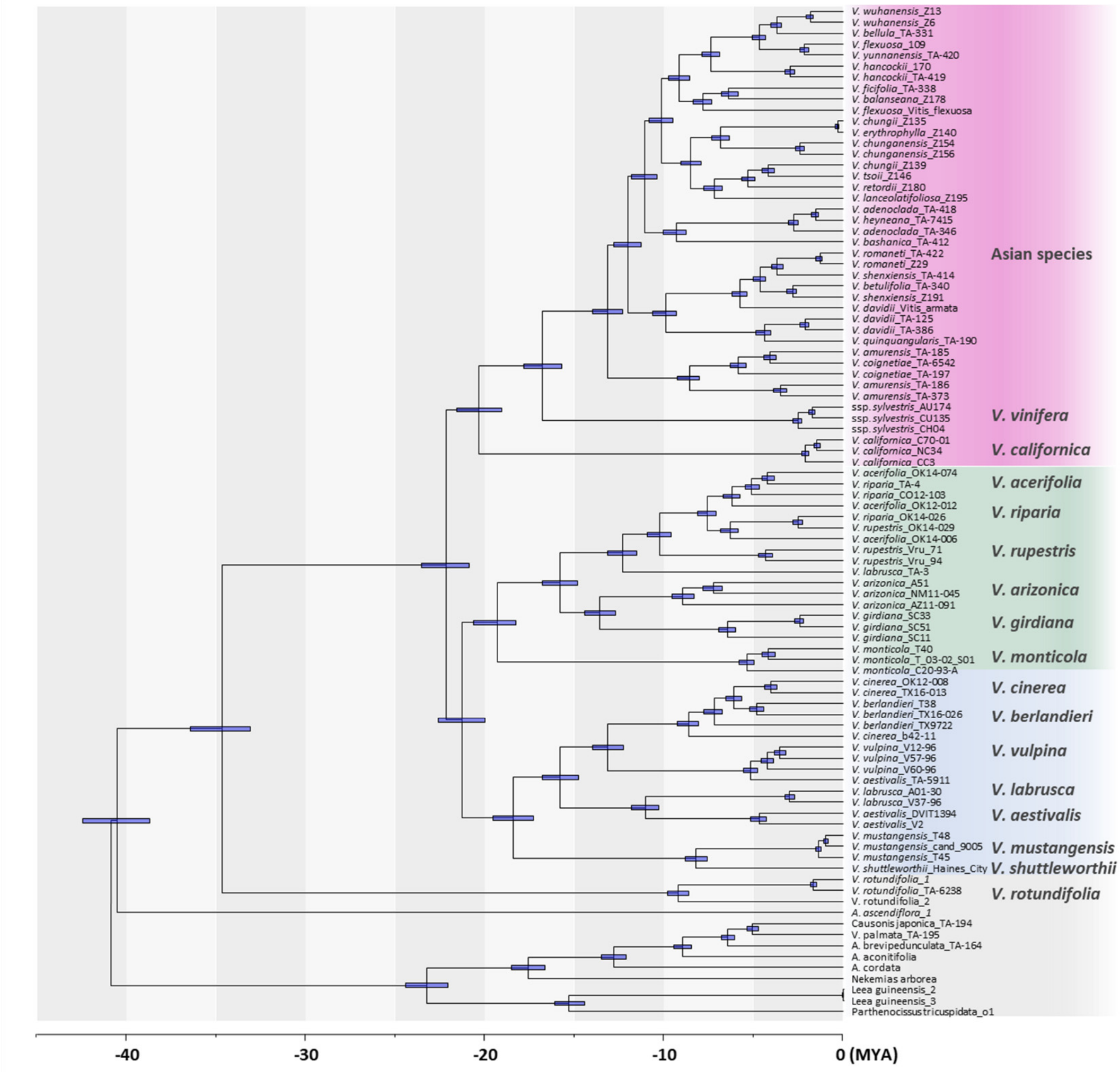

Supplementary Fig. 8. Time-calibrated phylogeny of non-hybrid *Vitis* species estimated using BEAST2. The chronogram was inferred using a relaxed molecular clock and a GTR nucleotide substitution model. Blue horizontal bars at the nodes represent the 95% Highest Posterior Density (HPD) intervals for the divergence time estimates. The scale axis at the bottom represents time in millions of years ago (Mya).

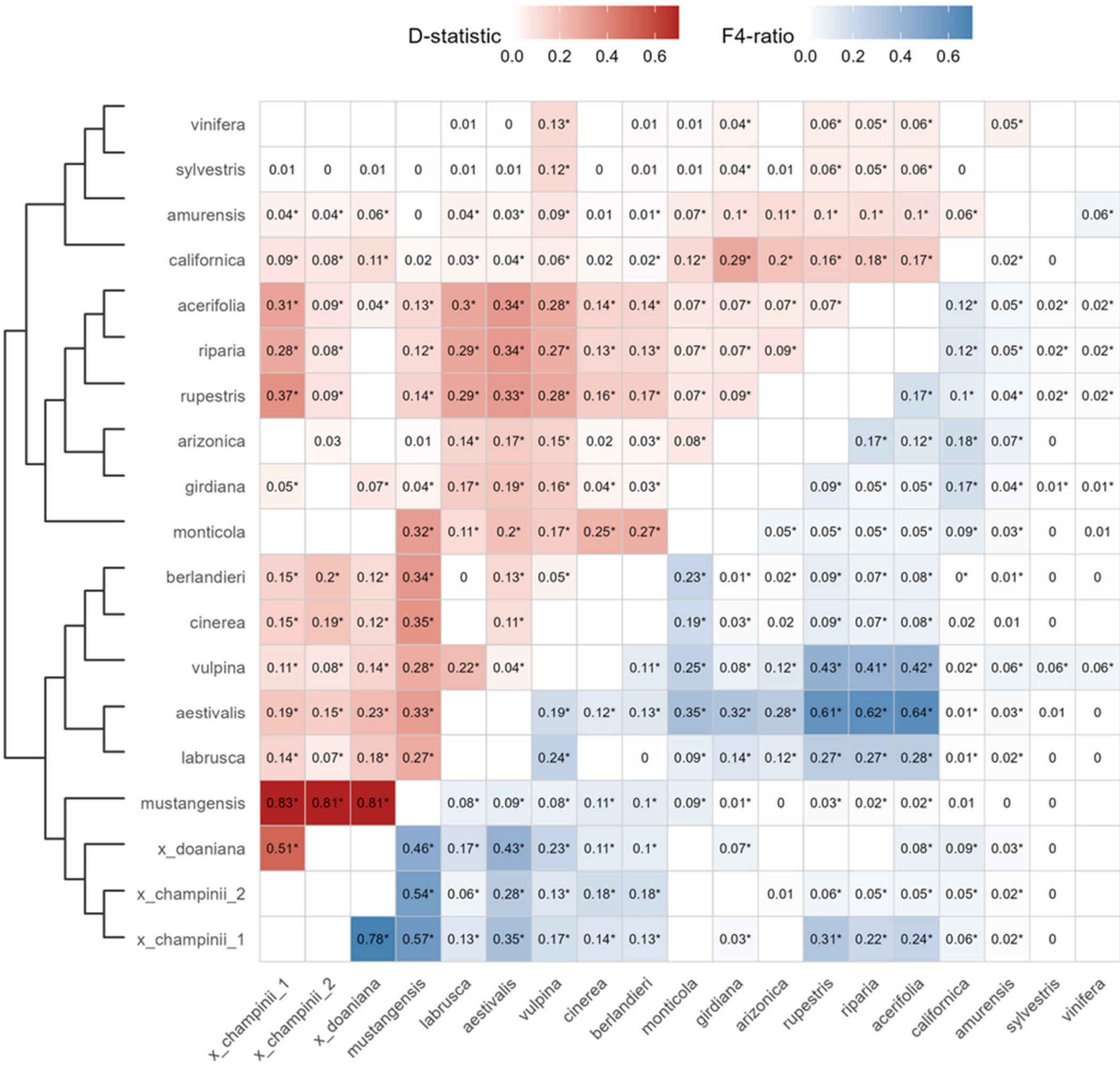

Supplementary Fig. 9. Genome-wide introgression signals from pairwise D-statistics and F4-ratio. Values marked with an asterisk denote significant introgression signals (Z-score > 4). Species are ordered according to the phylogeny shown on the left.

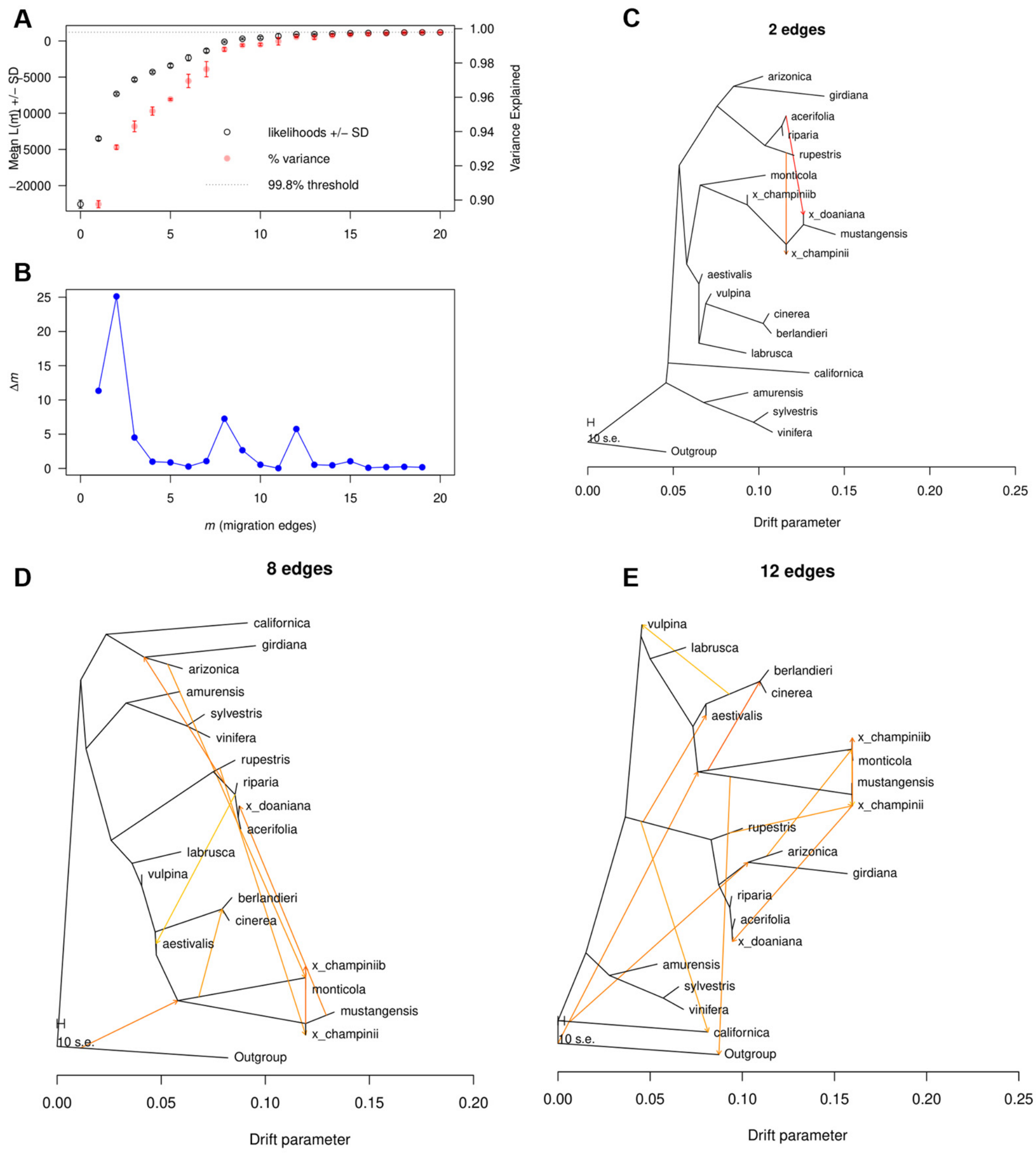

Supplementary Fig. 10. Gene flow events among different Vitis species estimated by Treemix and OptM. (A) The mean and standard deviation (SD) across 3 iterations for the composite likelihood (top axis) and proportion of variance (right axis) explained by models with 1-19 edges. **(B)** Distribution of deltaM statistic based on the second order rate of change in the likelihood with standard deviation considered. **(C-E)** Three models with different migration events that achieved >99.5% variance explained and high deltaM values.

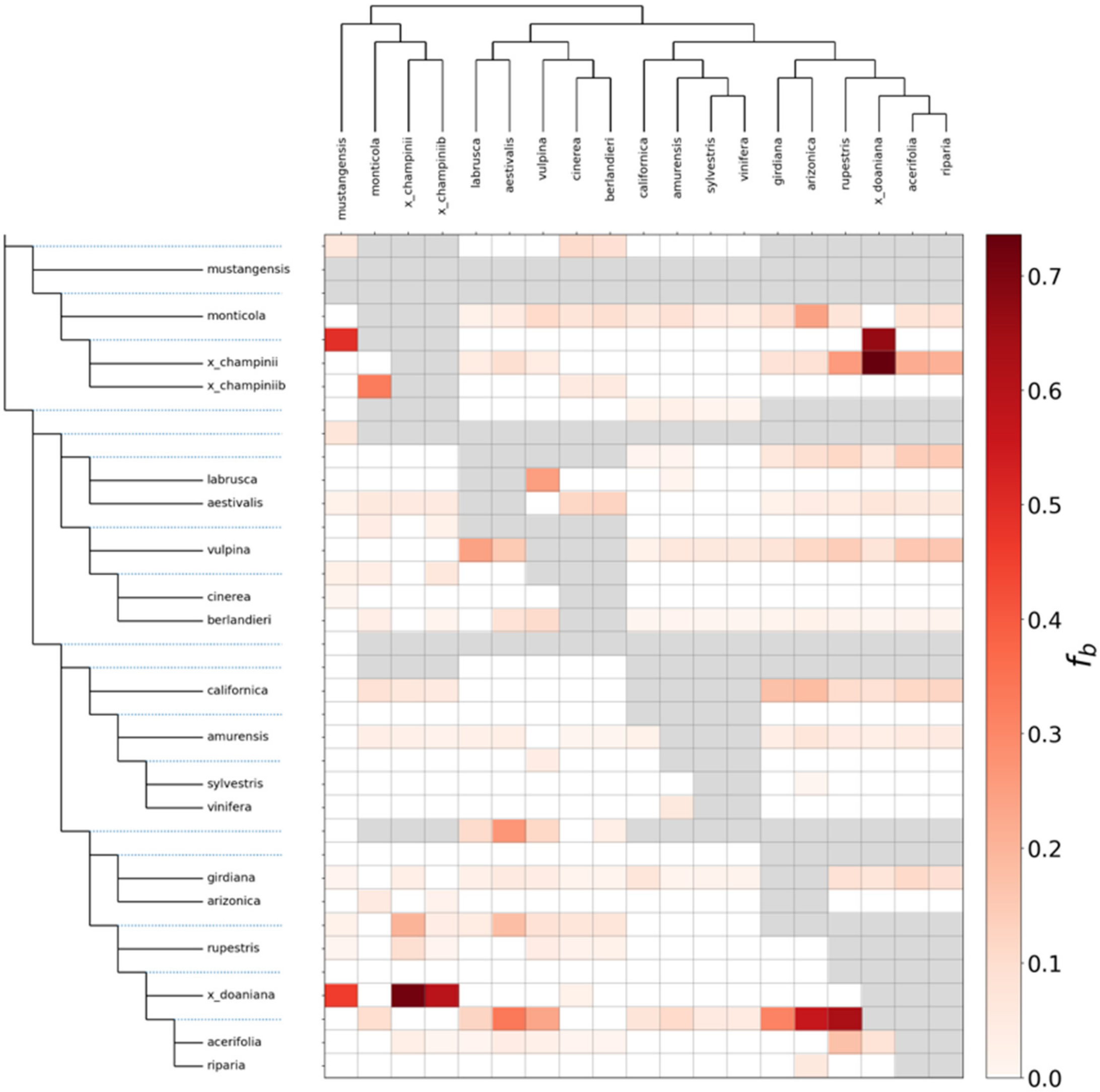

Supplementary Fig. 11. Global signals of introgression inferred by the f-branch (fb) statistic. The heatmap illustrates excess allele sharing between specific branches of the *Vitis* phylogeny (vertical axis) and potential donor species (horizontal axis). The phylogenetic tree on the left identifies the specific branch receiving the gene flow (indicated by dotted lines), while the tree on the top indicates the donor lineages.

Vitis.acerifolia

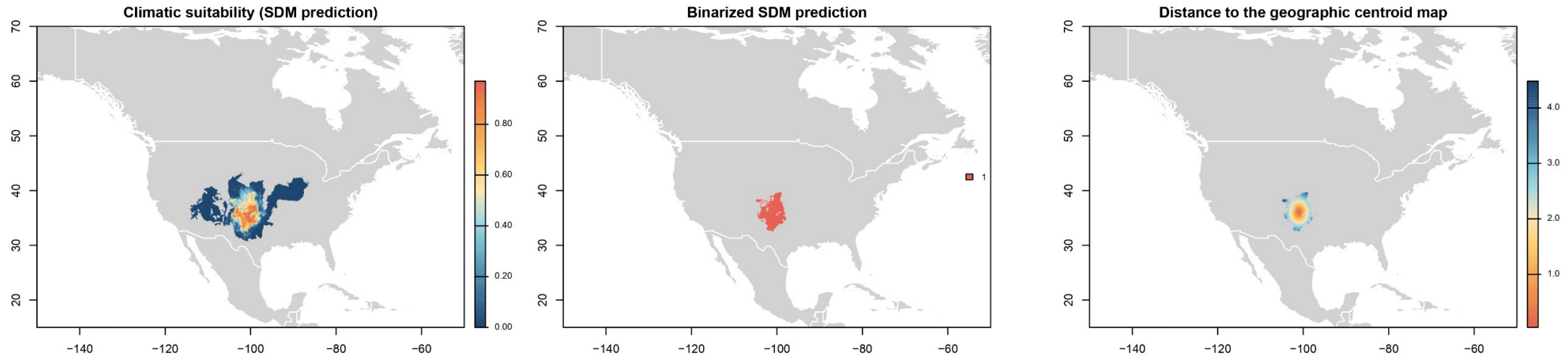

Vitis.aestivalis

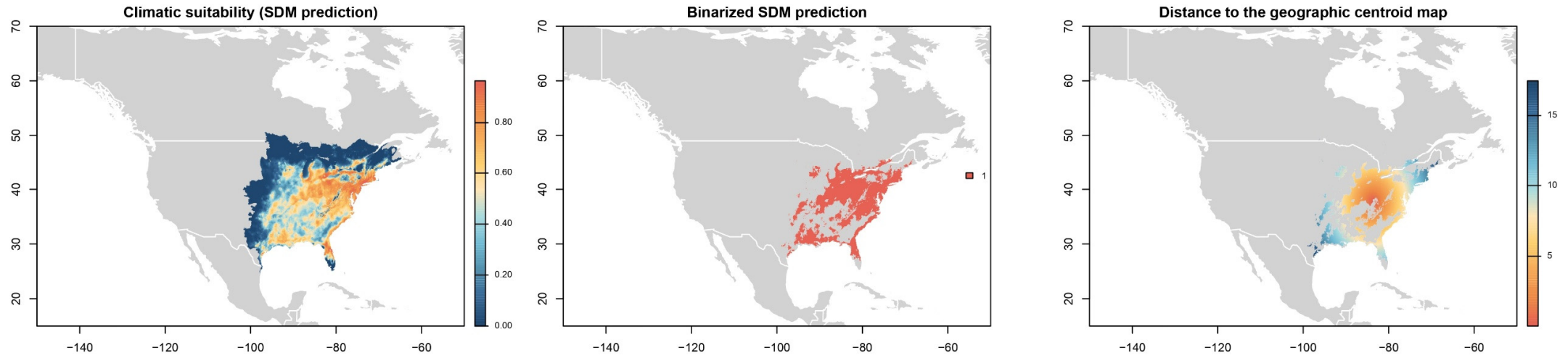

Vitis.arizonica

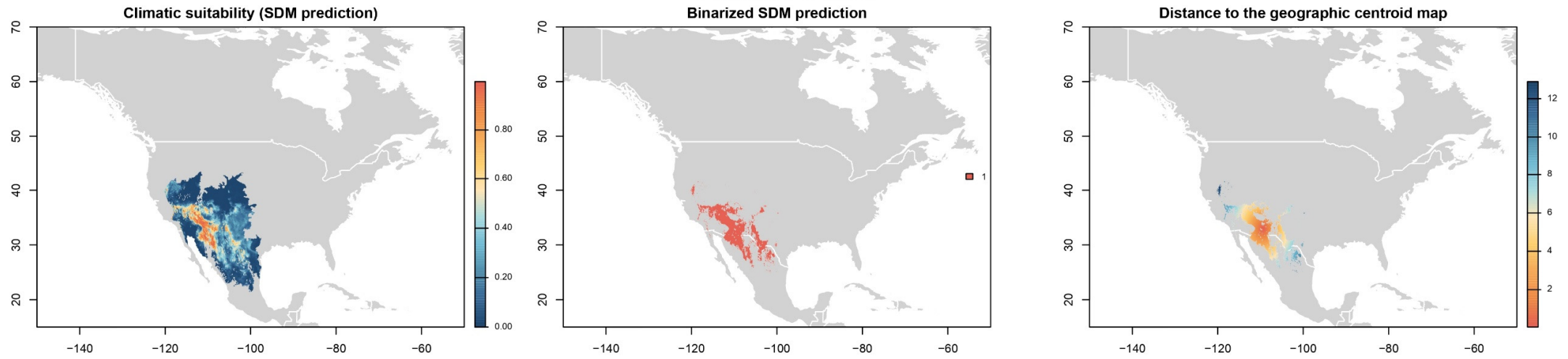

Vitis.californica

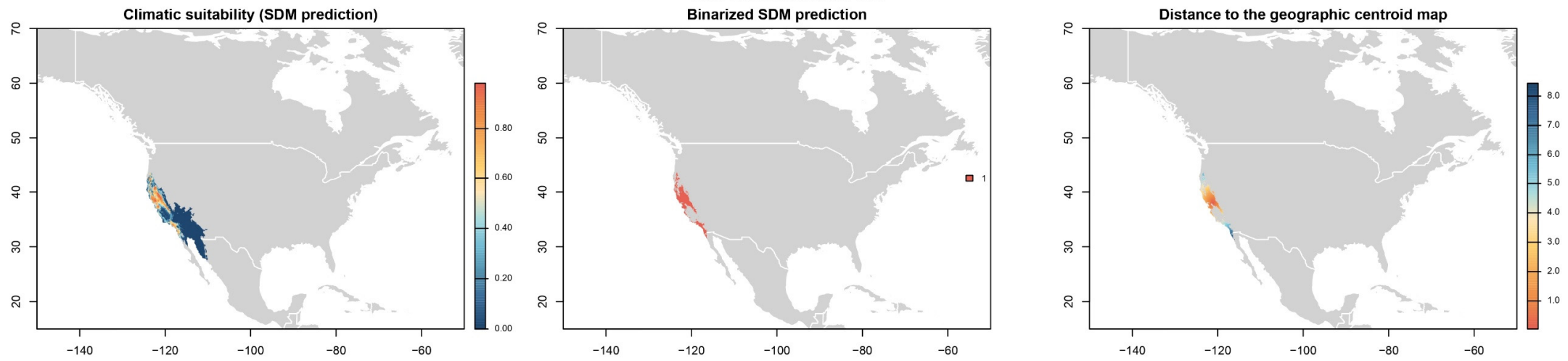

Vitis.cinerea

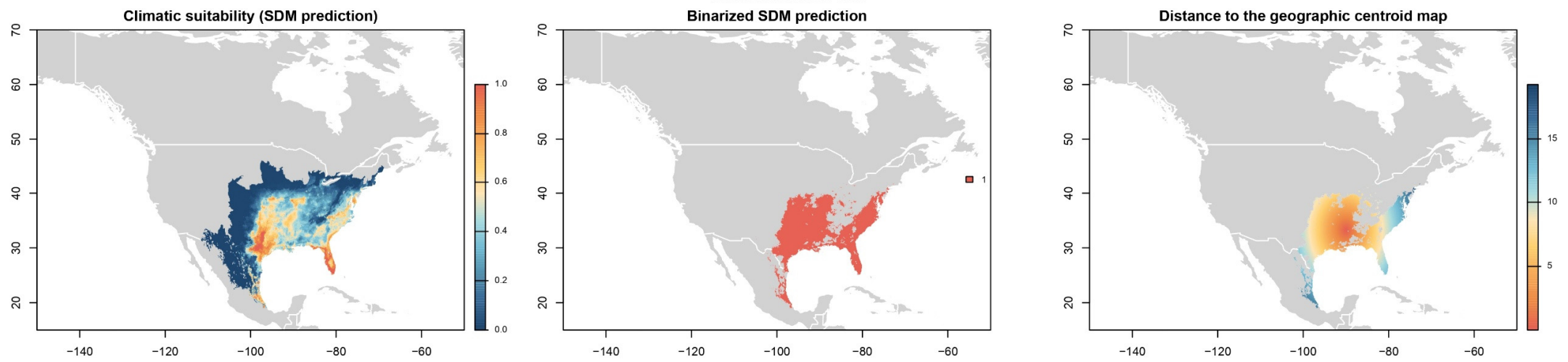

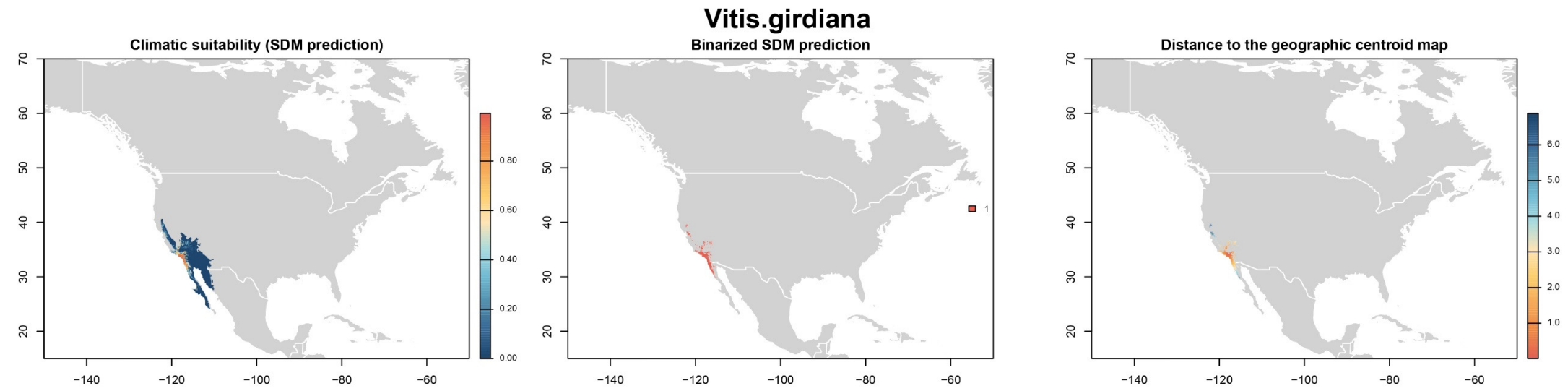
Vitis.girdiana
Climatic suitability (SDM prediction)
Binarized SDM prediction
Distance to the geographic centroid map

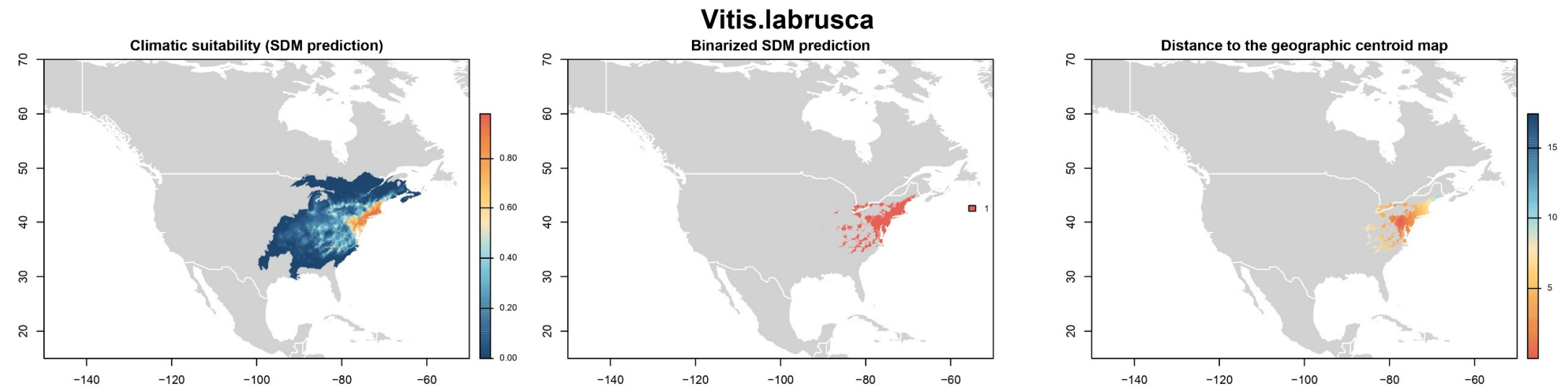
Vitis.labrusca
Climatic suitability (SDM prediction)
Binarized SDM prediction
Distance to the geographic centroid map

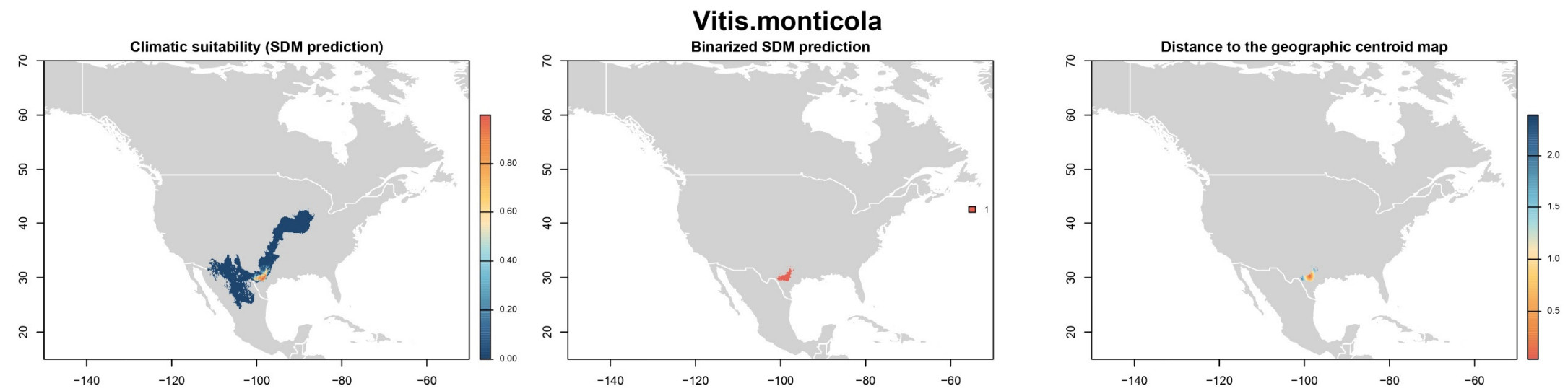
Vitis.monticola
Climatic suitability (SDM prediction)
Binarized SDM prediction
Distance to the geographic centroid map

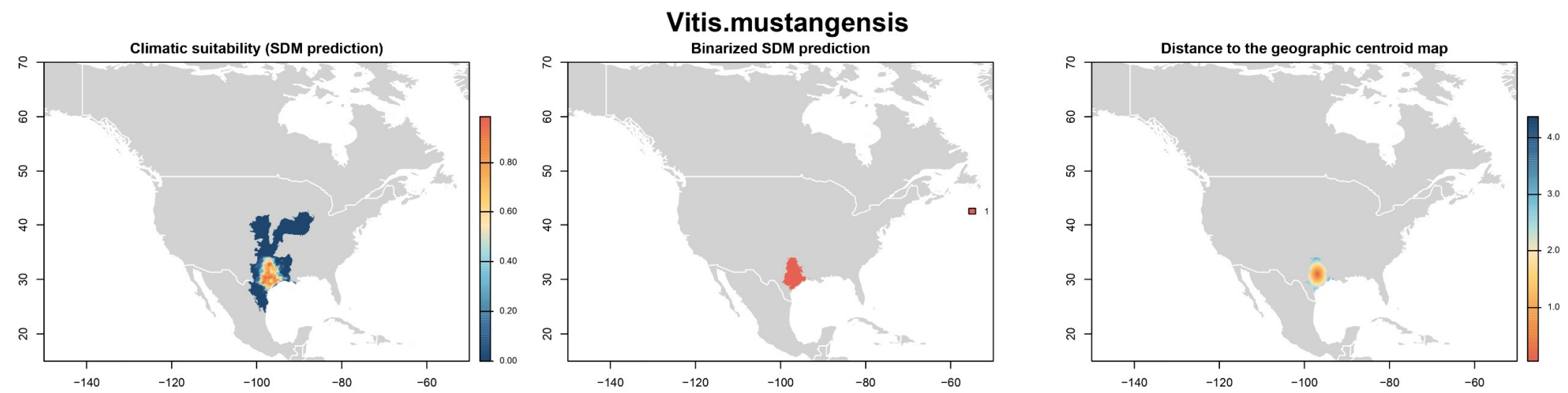
Vitis.mustangensis
Climatic suitability (SDM prediction)
Binarized SDM prediction
Distance to the geographic centroid map

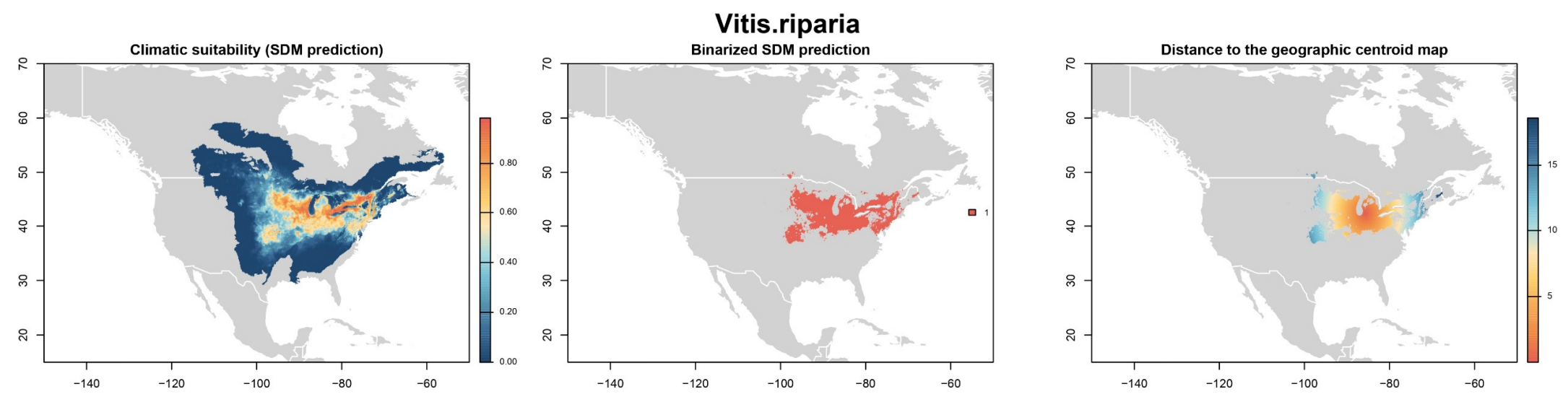
Vitis.riparia
Climatic suitability (SDM prediction)
Binarized SDM prediction
Distance to the geographic centroid map

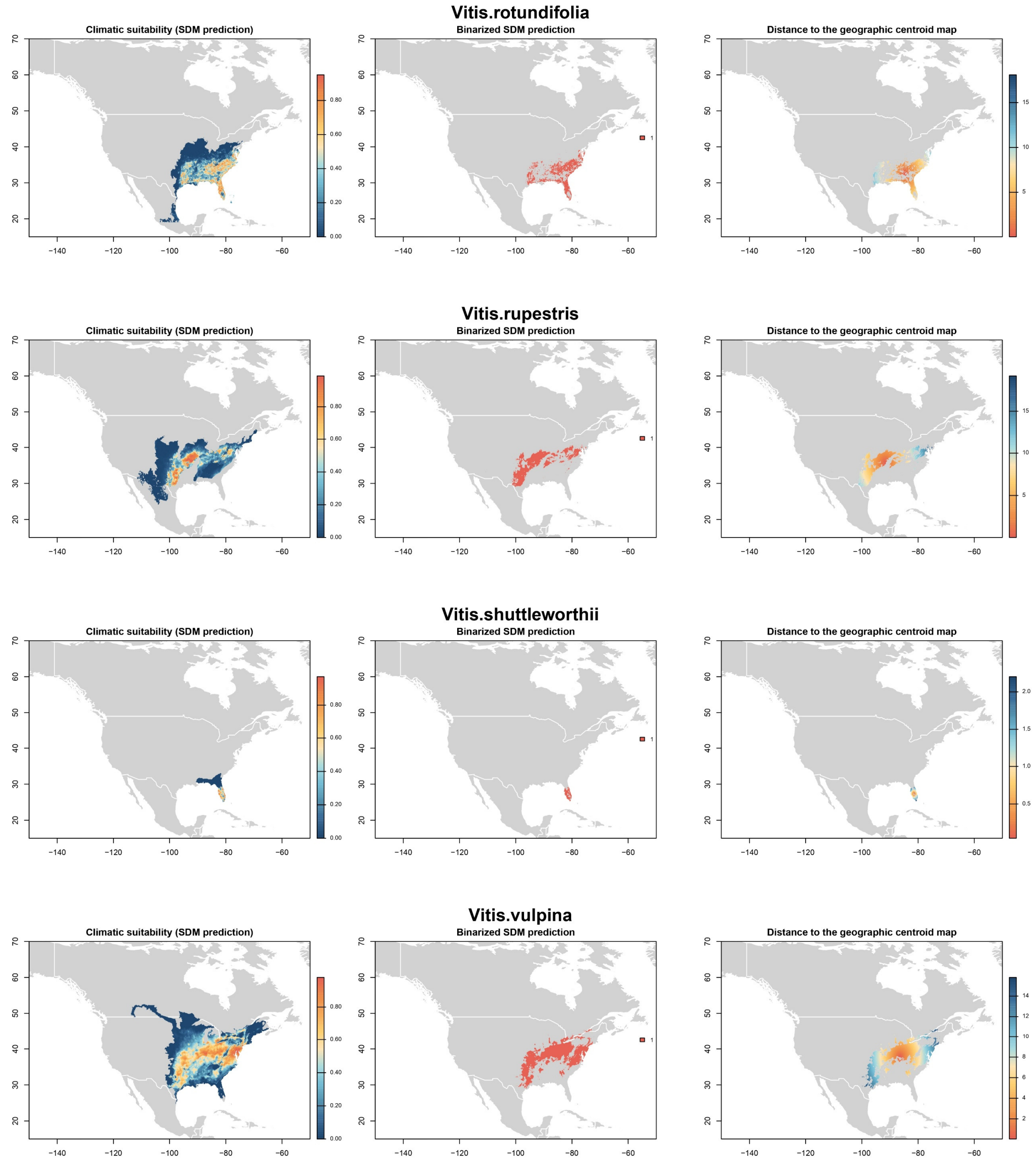

Supplementary Fig. 12. Species Distribution Models (SDMs) for 14 North American Vitis species. Left, map displaying the probability of occurrence across the accessible area defined by biogeographic barriers (M), with warmer colors indicating higher probability; Middle, binarized map derived from the SDM probability values using TSS and ROC thresholds; Right, map showing for each location the distance to the geographic centroid of the binarized map, with warmer colors showing a lower distance to the geographic centroid. All maps are based on bioclimatic variables from 1970 to 2000 (see Methods).

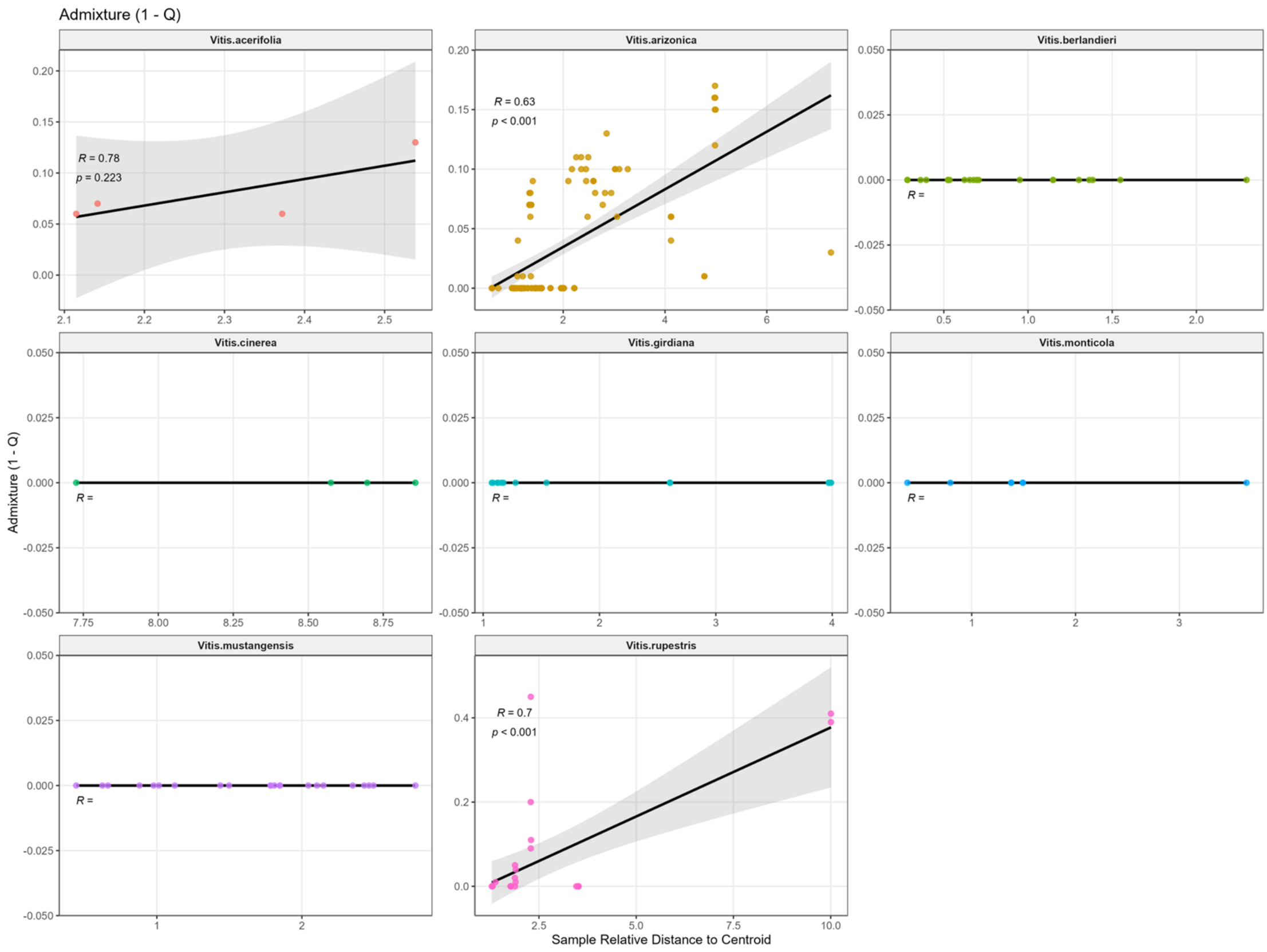

Supplementary Fig. 13. Within-species correlations between the geographic distance to the niche centroid and genomic admixture levels. For each *Vitis* species, points represent sampled individuals plotted by their relative geographic distance to the species niche centroid (x-axis) and their admixture level (y-axis). For each individual, admixture was quantified as 1−Q, where Q is the individual's maximum ancestry proportion inferred from the population structure analysis at K=13.

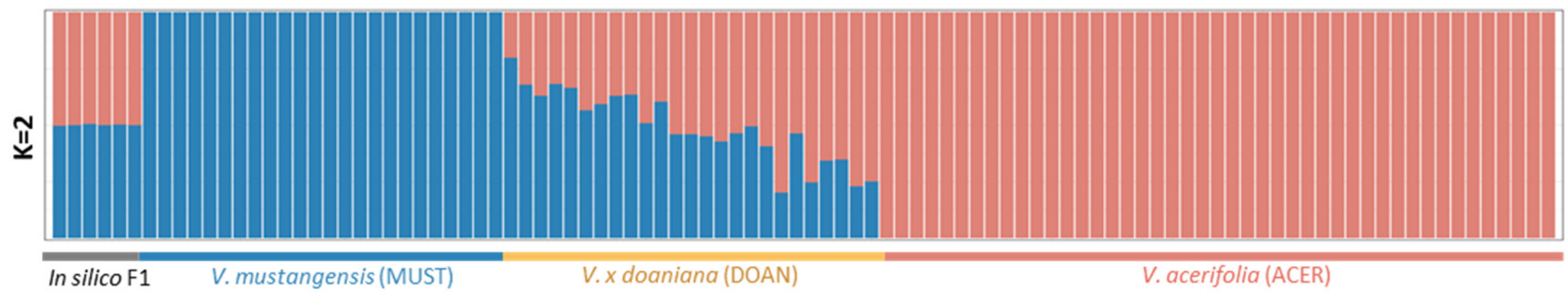

Supplementary Fig. 14. Global ancestry proportions for the *V. x doaniana* hybrid group and its putative parents. Model-based admixture analysis (K=2) showing global ancestry proportions for *V. x doaniana* individuals, individuals from the hypothesized parents (*V. mustangensis* and *V. acerfolia*), and *in silico* F1 individuals.

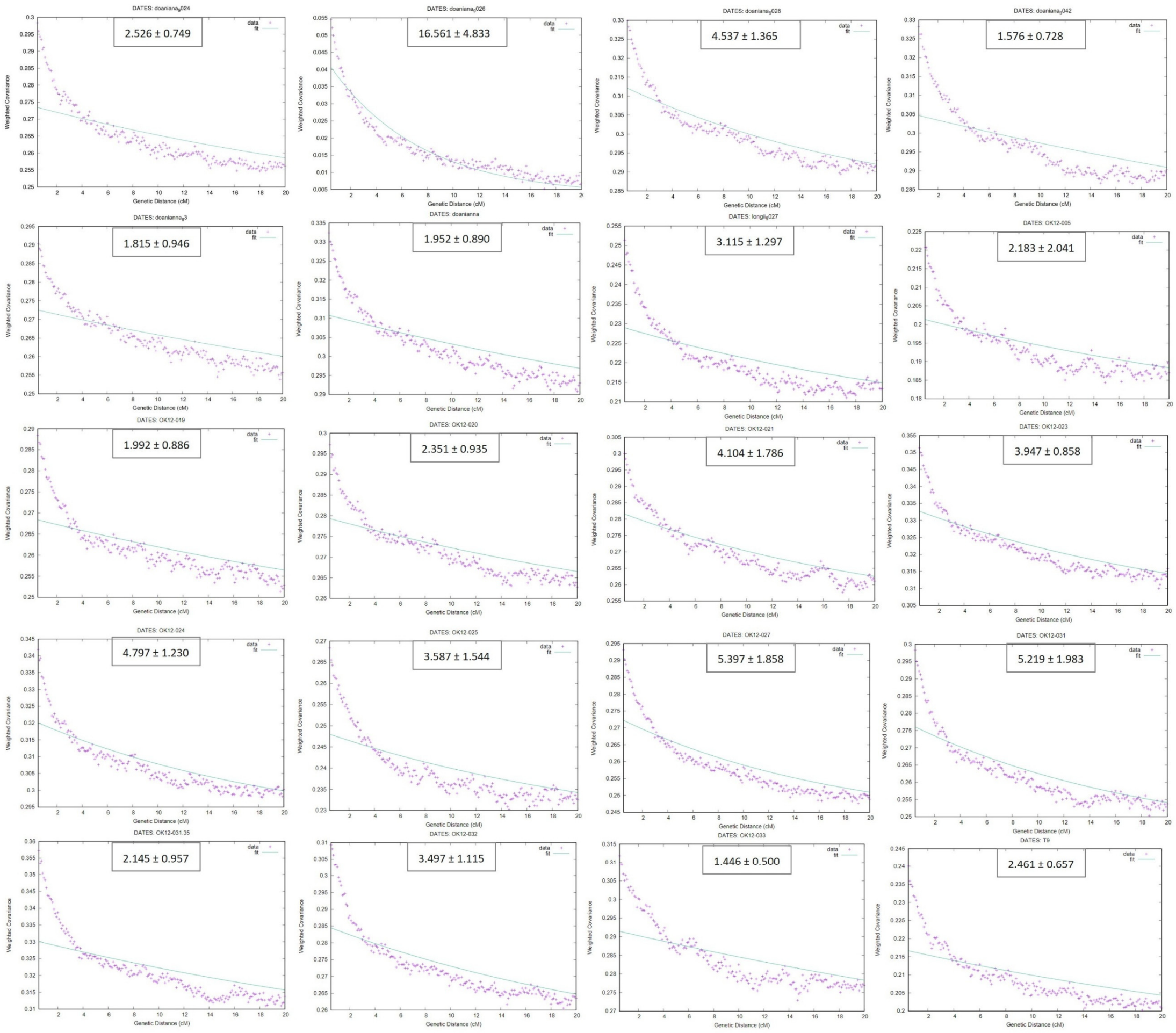

Supplementary Fig. 15. Inference of hybridization timing for *V. doaniana* individuals using DATES. The panels display the decay of weighted ancestry covariance (y-axis) as a function of genetic distance in centimorgans (cM, x-axis). The green line represents the fitted exponential decay curve used to estimate the time since admixture. The inferred number of generations since hybridization (mean +- standard error) is indicated below each plot. Each graph represents a separate individual.

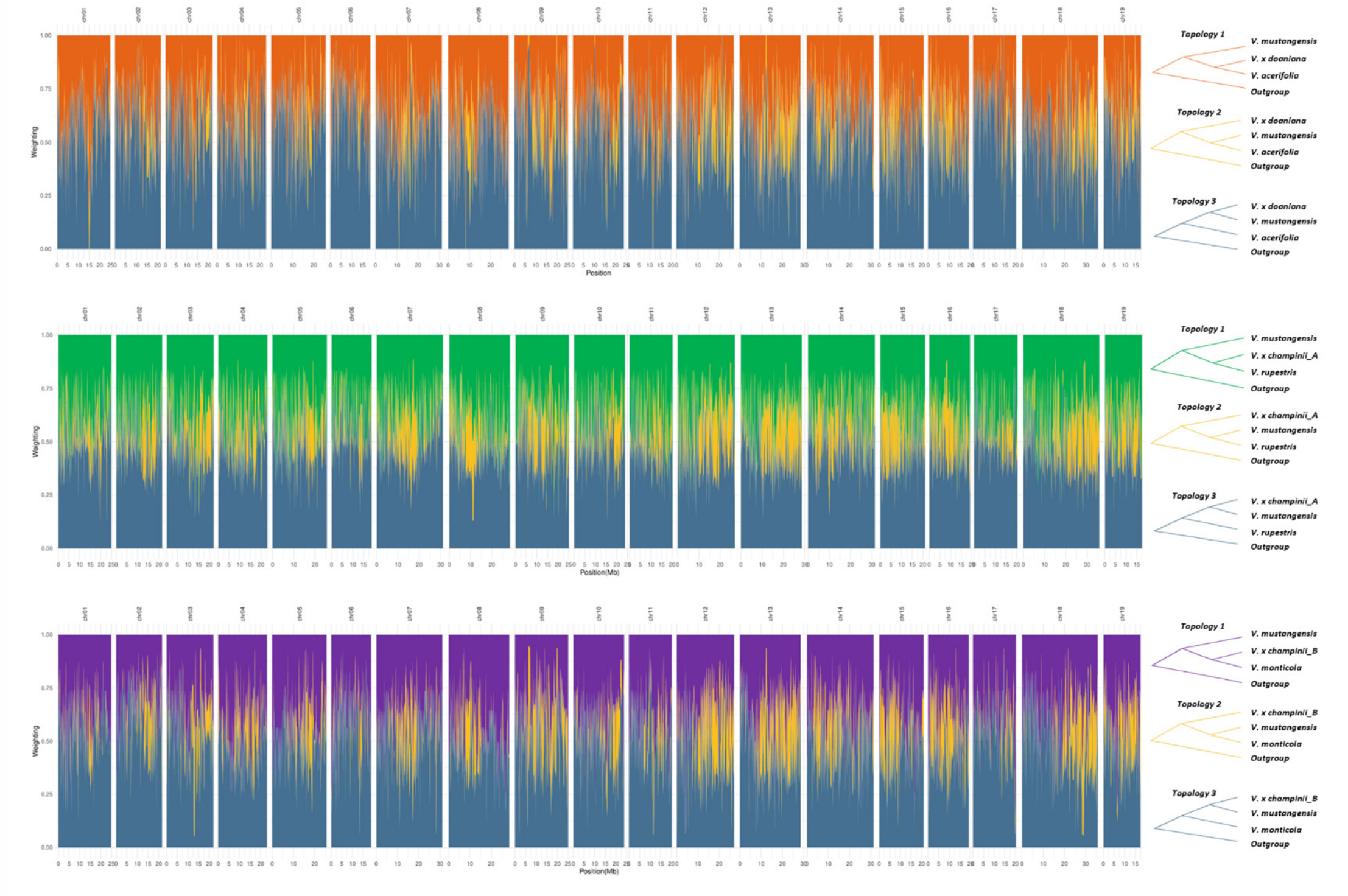

Supplementary Fig. 16. TWISST analysis of topology weighting across the genome for hybrid groups and their putative parental species. Topology weighting was estimated across sliding genomic windows for three four-taxon comparisons. For each comparison, the three possible rooted topologies are shown at right, and colored traces indicate their relative support across genomic windows along each chromosome.

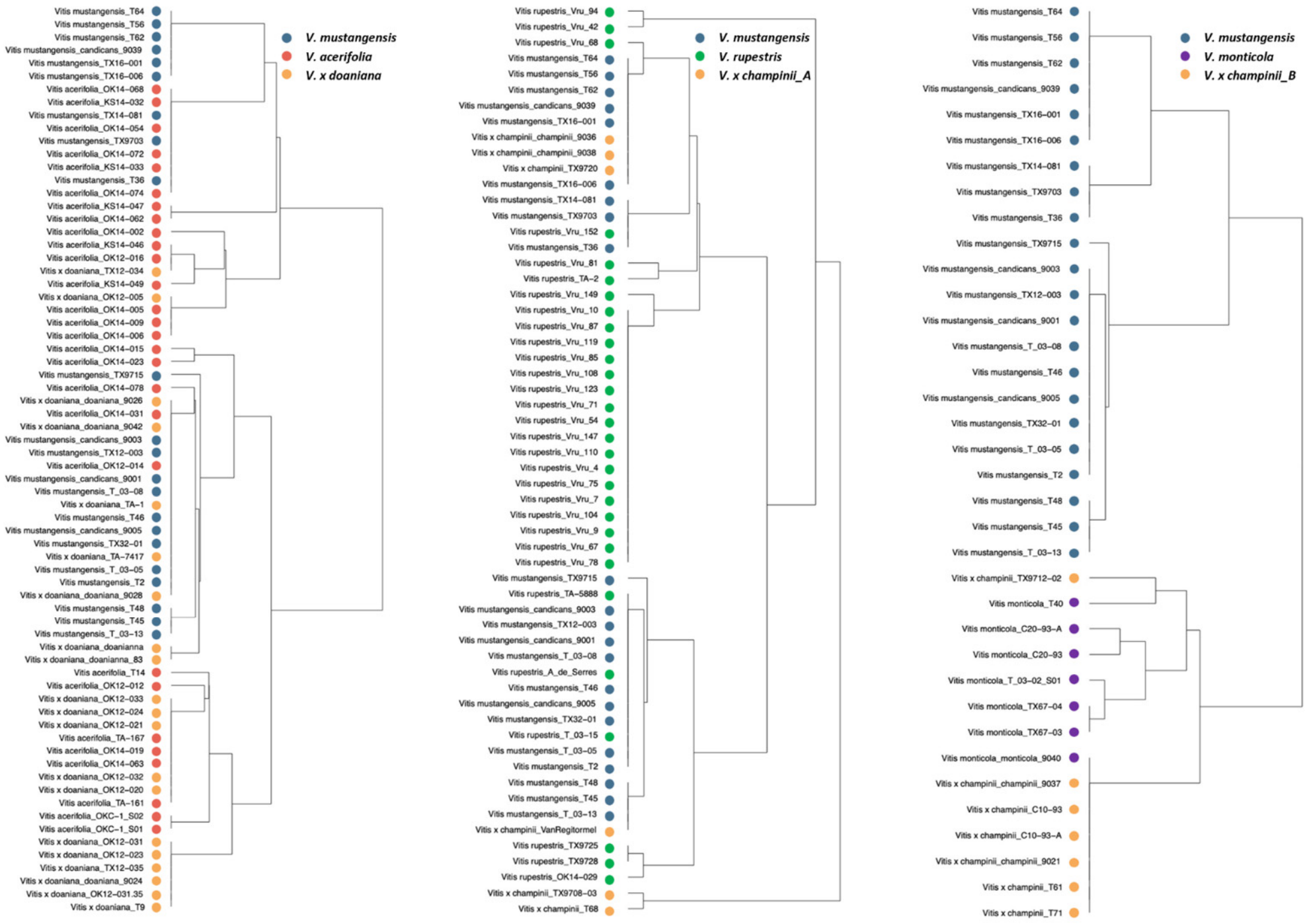

Supplementary Fig. 17. Chloroplast haplotype phylogenies for hybrid Vitis groups and their putative parental species. Plastid trees are shown for *V.* × *doaniana* with *V. mustangensis* and *V. acerifolia* (left), *V.* × *champinii* group A with *V. mustangensis* and *V. rupestris* (center), and *V.* × *champinii* group B with *V. mustangensis* and *V. monticola* (right). Colored circles indicate species. In all three comparisons, hybrid individuals do not form a monophyletic plastid lineage, but instead cluster with different putative parental taxa.

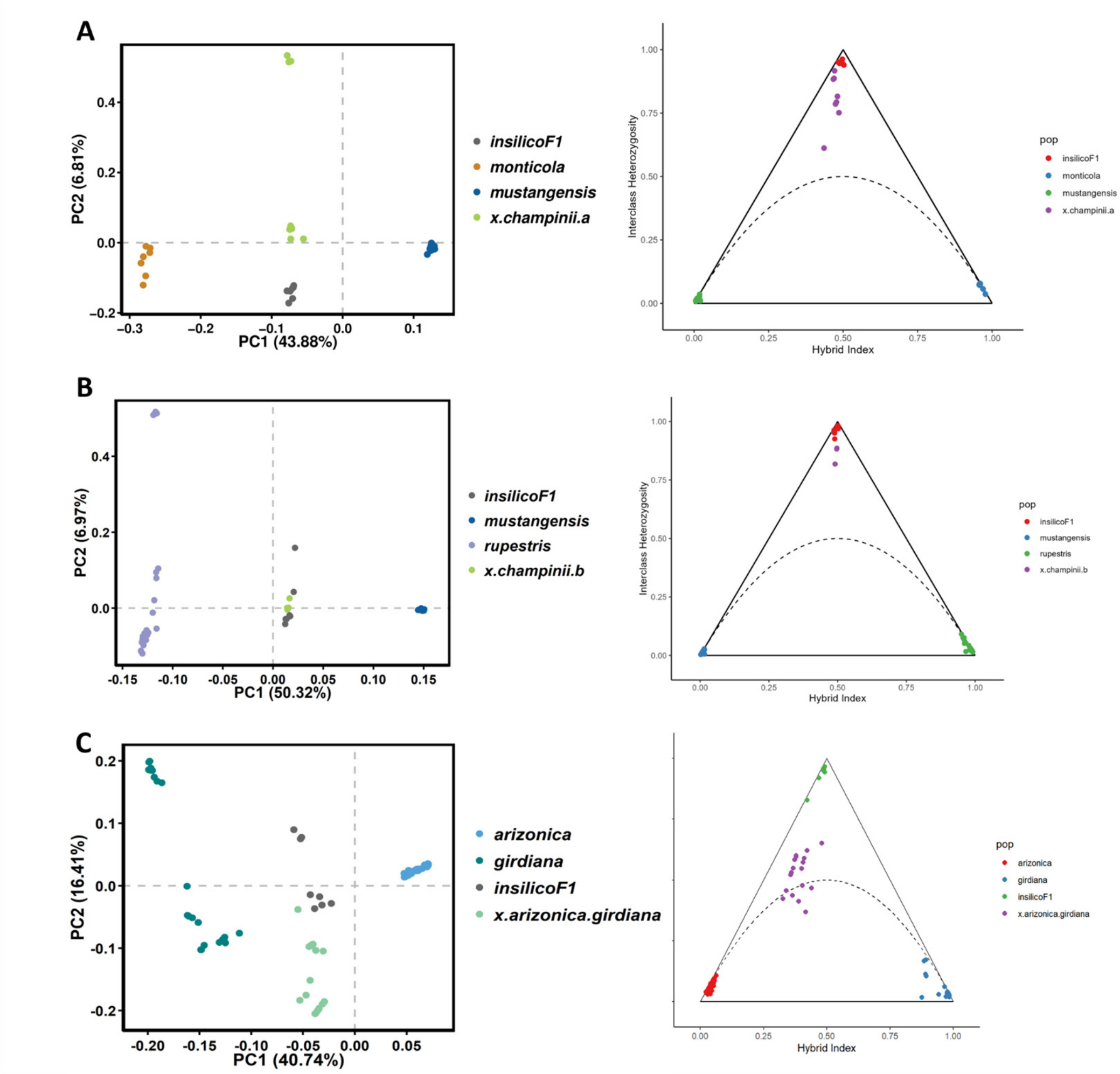

Supplementary Fig. 18. Genomic evidence for recent hybridization in *V. × champinii* lineages and *V. arizonica/girdiana* hybrids. For each putative hybrid group (rows), the left panel shows a principal component analysis (PCA) including the two inferred parental taxa and an in silico F1 control (constructed from parental genotypes). In all cases, putative hybrid individuals cluster near the in silico F1s and occupy intermediate positions between parental clusters along PC1. The right panel shows triangular (hybrid-index vs. interclass heterozygosity) plots. Putative hybrid individuals plot close to the apex and along the upper region of the triangle, consistent with very recent hybrid ancestry (F1/F2 and early backcross generations).

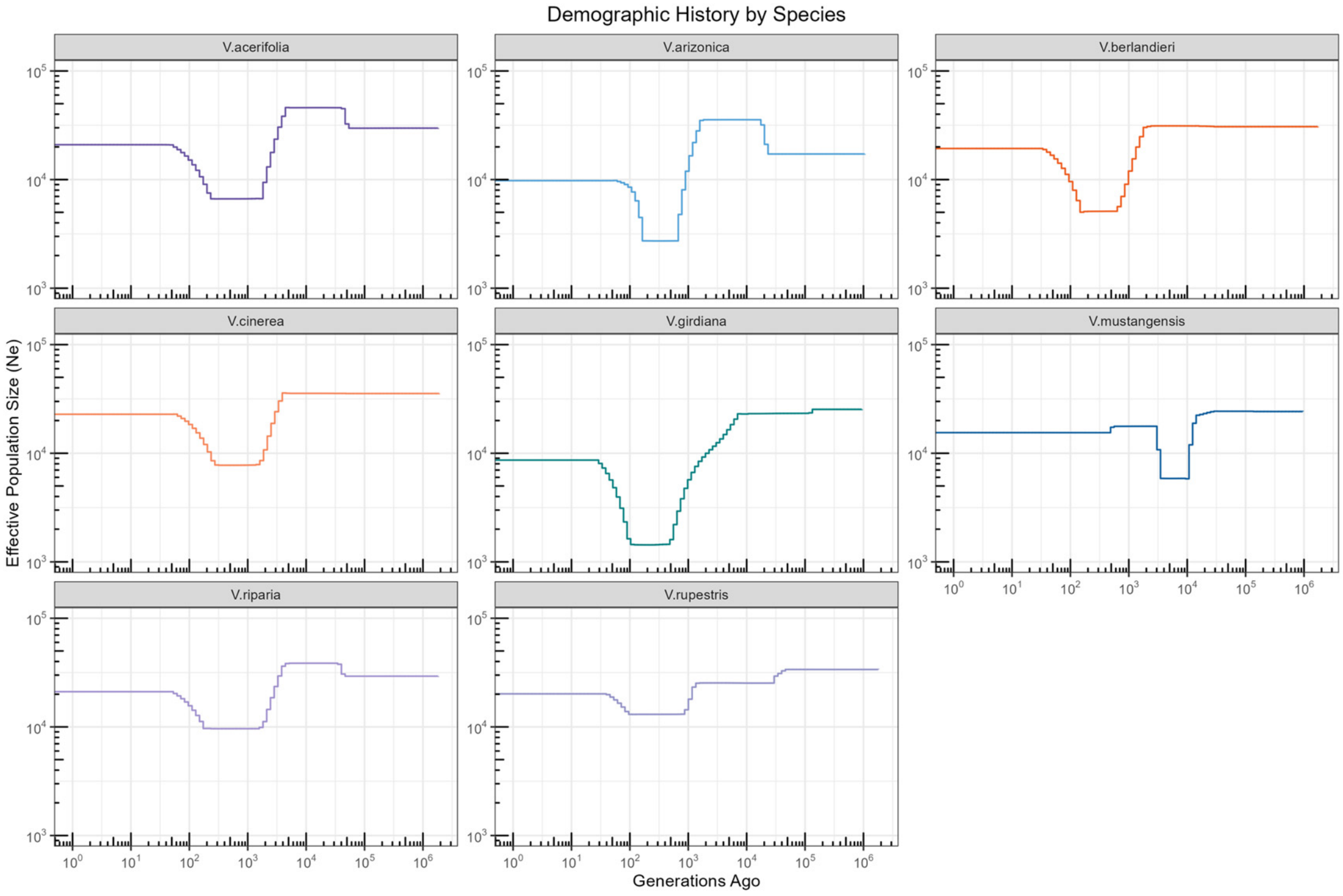

Supplementary Fig. 19. Demographic history of each Vitis species inferred by mushi. Generation estimates were inferred by assuming a generation time of 3 years and mutation rates of 5.4e-9 per site per generation, respectively. The panels display the changes in effective population size over time (generations ago) for selected Vitis species. The trajectories were reconstructed from the unfolded site frequency spectrum (SFS) using mushi v0.2. Both axes are plotted on a logarithmic scale.

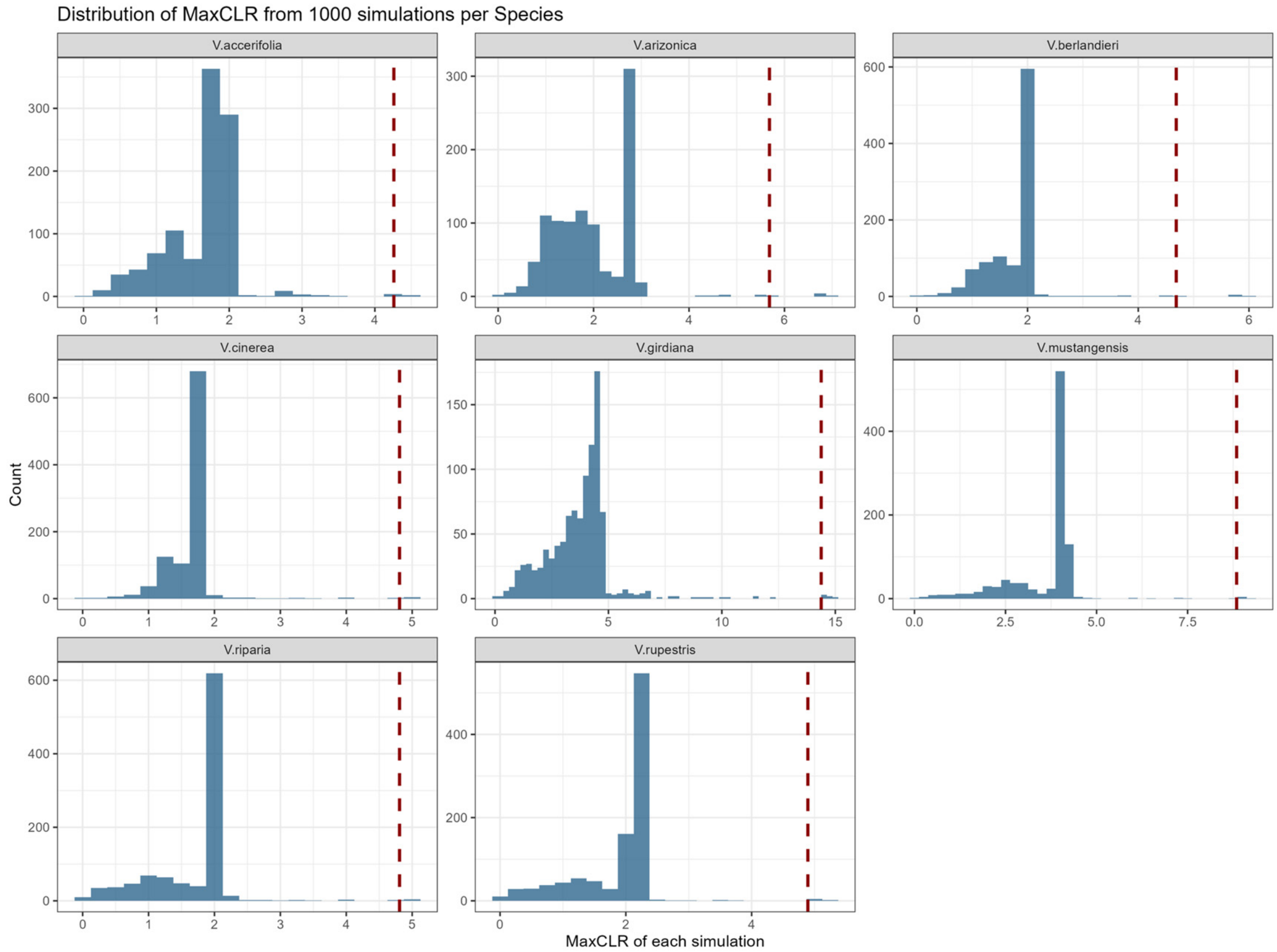

Supplementary Fig. 20. Null distributions of the maximum Composite Likelihood Ratio (CLR) statistic derived from 1000 neutral coalescent simulations. For each of the eight analyzed Vitis species, the histogram displays the distribution of the maximum CLR value obtained from 1,000 neutral simulations generated using msprime under estimated demographic histories. The vertical red dashed line marks the 99.5th percentile of each distribution, which served as the threshold for identifying selective sweeps in the empirical genomic data.

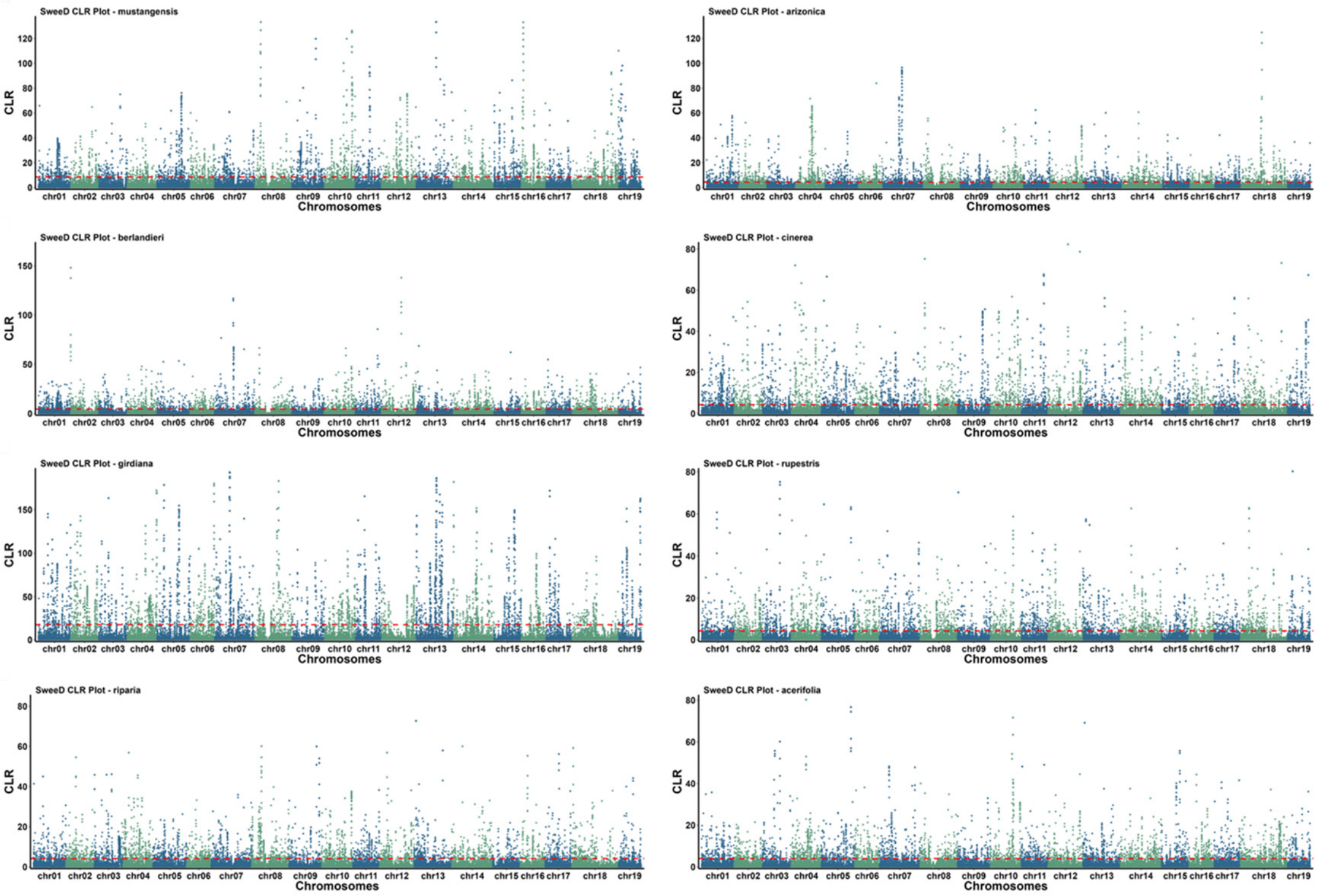

Supplementary Fig. 21. Genome-wide scans for selective sweeps across eight North American *Vitis* species. The Manhattan plots display the Composite Likelihood Ratio (CLR) statistic inferred by SweeD (y-axis) along the 19 chromosomes (x-axis).

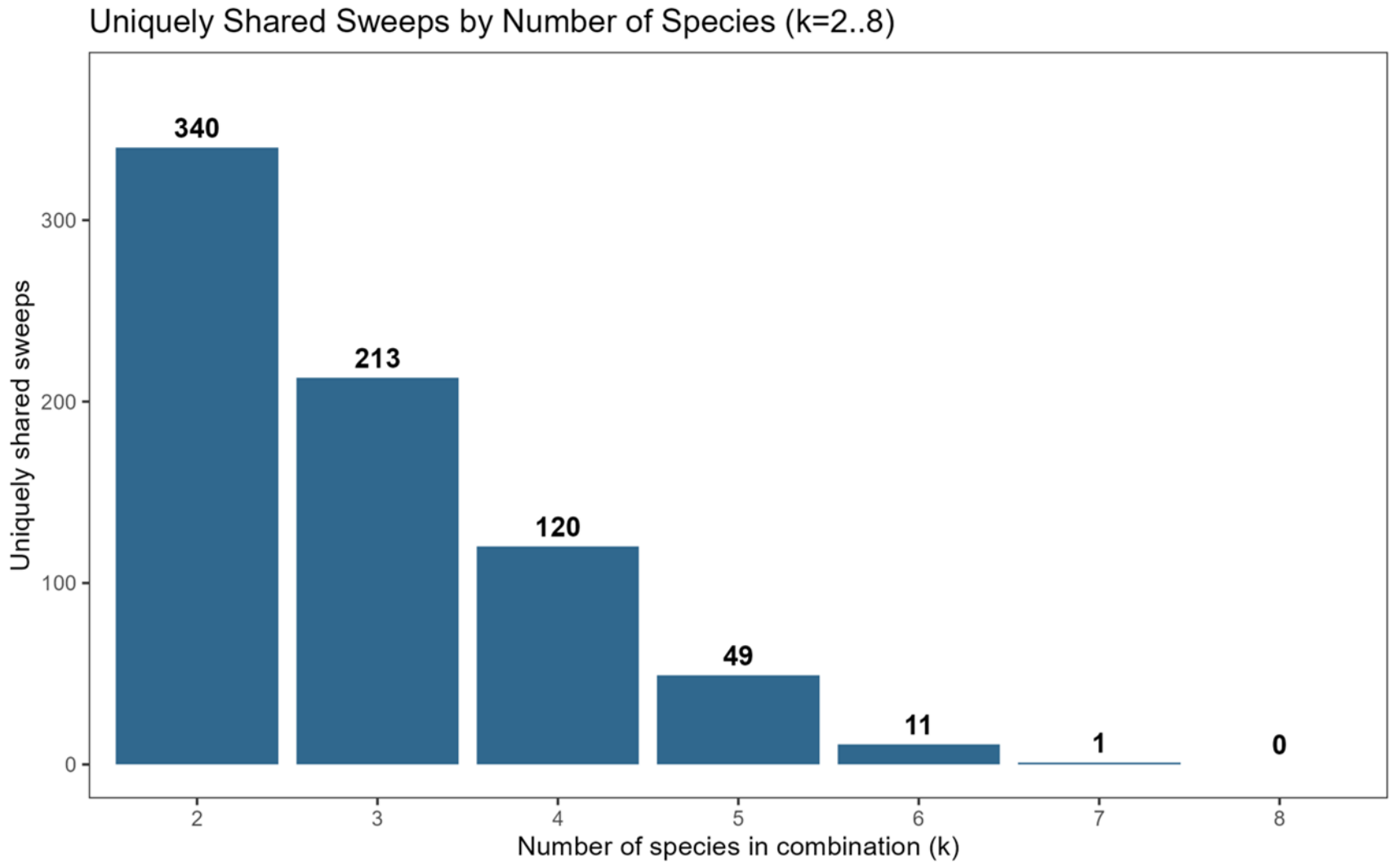

Supplementary Fig. 22. Distribution of shared selective sweeps across multiple Vitis species. The bar chart displays the number of selective sweeps shared by varying numbers of species, ranging from pairs (n=2) to sweeps shared across all eight analyzed species (n=8). A selective sweep was defined as shared if the identified genomic interval in one species overlapped by at least 60% of the sweep length in another species.

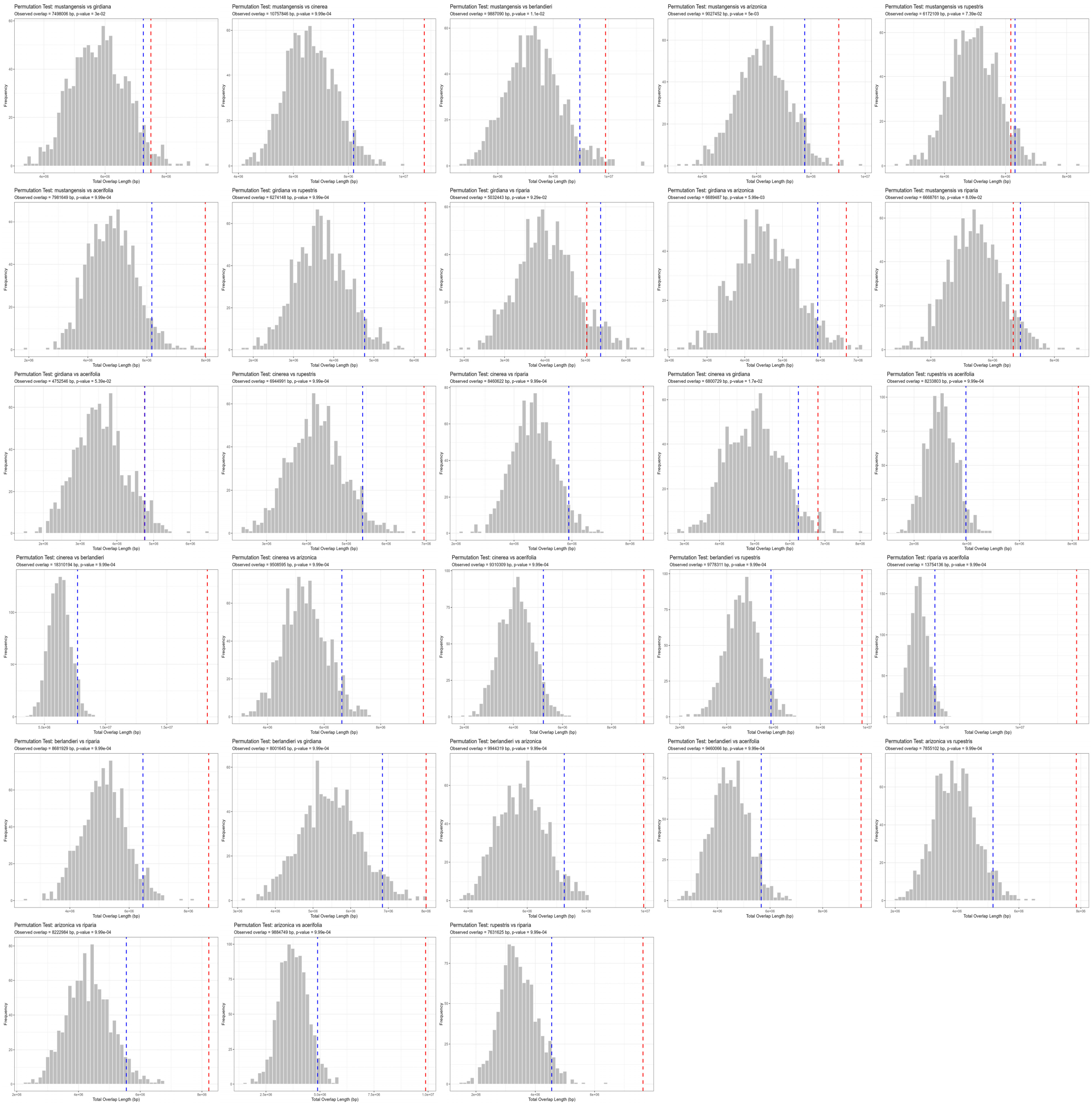

Supplementary Fig. 23. Permutation tests for shared sweeps among Vitis species. For each species pair, the histogram shows the null distribution of total sweep-overlap length (bp) expected under randomization. The red dashed line marks the observed total overlap length between the two species. The blue dashed line marks the 95th percentile of the null distribution. Observed values to the right of the blue line indicate significantly greater overlap than expected by chance.

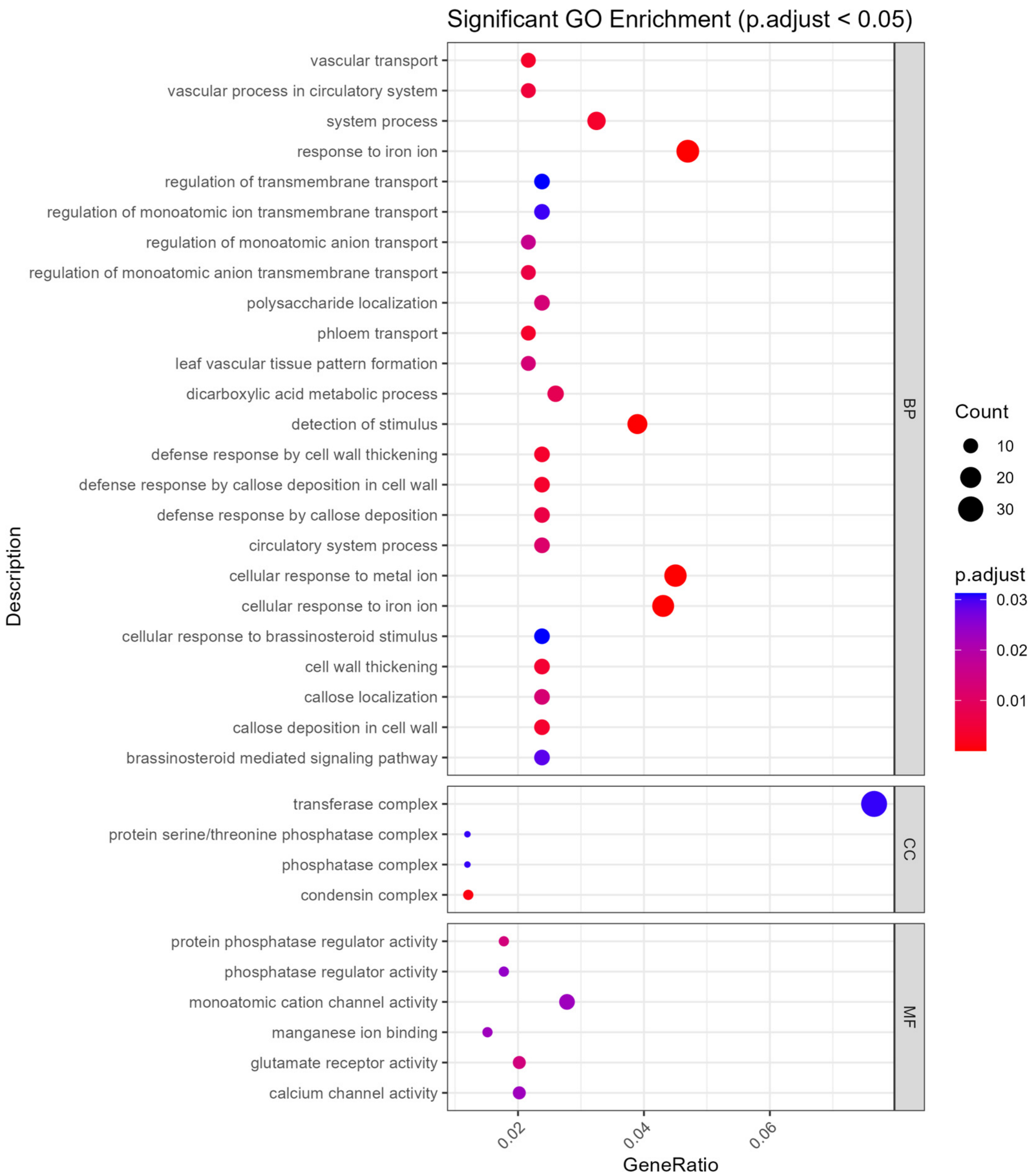

Supplementary Fig. 24. Gene Ontology (GO) enrichment of selective sweeps among Vitis species. The dot plot displays significantly enriched GO terms (P.adjust < 0.05) identified within selective sweep regions across eight *Vitis* species. Enriched terms are grouped by GO category: biological process (BP), cellular component (CC), and molecular function (MF).
